# Neuroinflammation in neurodegenerative diseases: Focusing on the mediation of T lymphocytes

**DOI:** 10.4103/NRR.NRR-D-24-01539

**Published:** 2025-06-19

**Authors:** Ke Li, Rongsha Chen, Ruohua Wang, Wenhui Fan, Ninghui Zhao, Zhongshan Yang, Jinyuan Yan

**Affiliations:** 1 Experiment Center, The Second Affiliated Hospital of Kunming Medical University, Kunming, Yunnan Province, China; 2 Department of Blood Transfusion, Yunnan Cancer Hospital, Kunming, Yunnan Province, China; 3 Department of Neurosurgery, The Second Affiliated Hospital of Kunming Medical University, Kunming, Yunnan Province, China; 4 Yunnan Provincial Key Laboratory of Molecular Biology for Sino Medicine, Yunnan University of Traditional Chinese Medicine, Kunming, Yunnan Province, China

**Keywords:** Alzheimer’s disease, amyotrophic lateral sclerosis, CD4^+^ T cell, CD8^+^ T cell, helper T cell, multiple sclerosis, neurodegenerative diseases, neuroinflammation, Parkinson’s disease, regulatory T cell

## Abstract

Neurodegenerative diseases are a group of illnesses characterized by the gradual deterioration of the central nervous system, leading to a decline in patients’ cognitive, motor, and emotional abilities. Neuroinflammation plays a significant role in the progression of these diseases. However, there is limited research on therapeutic approaches to specifically target neuroinflammation. The role of T lymphocytes, which are crucial mediators of the adaptive immune response, in neurodegenerative diseases has been increasingly recognized. This review focuses on the involvement of T lymphocytes in the neuroinflammation associated with neurodegenerative diseases. The pathogenesis of neurodegenerative diseases is complex, involving multiple mechanisms and pathways that contribute to the gradual degeneration of neurons, and T cells are a key component of these processes. One of the primary factors driving neuroinflammation in neurodegenerative diseases is the infiltration of T cells and other neuroimmune cells, including microglia, astrocytes, B cells, and natural killer cells. Different subsets of CD4^+^ T cells, such as Th1, Th2, Th17, and regulatory T cells, can differentiate into various cell types and perform distinct roles within the neuroinflammatory environment of neurodegenerative diseases. Additionally, CD8^+^ T cells, which can directly regulate immune responses and kill target cells, also play several important roles in neurodegenerative diseases. Clinical trials investigating targeted T cell therapies for neurodegenerative diseases have shown that, while some patients respond positively, others may not respond as well and may even experience adverse effects. Targeting T cells precisely is challenging due to the complexity of immune responses in the central nervous system, which can lead to undesirable side effects. However, with new insights into the pathophysiology of neurodegenerative diseases, there is hope for the establishment of a solid theoretical foundation upon which innovative treatment strategies that target T cells can be developed in the future.

## Introduction

Neurodegenerative diseases (NDD) are a group of disorders that affect the brain and nervous system, typically characterized by the progressive loss and dysfunction of neurons. Currently, there are no known cures for these conditions, and they often worsen over time. NDD can result in a wide range of symptoms that adversely impact motor, cognitive, sensory, and autonomic nervous system functions. Common NDD include Parkinson’s disease (PD), Alzheimer’s disease (AD), amyotrophic lateral sclerosis (ALS), and multiple sclerosis (MS). Although the exact causes of NDD remain unknown, neuroinflammation is a significant factor in their pathophysiology (Han et al., 2024). Neuroinflammation continuously challenges neurons, leading to neurodegeneration. Therefore, interventions targeting neuroinflammation may help reduce neuronal death or promote neuronal survival, ultimately providing protection against neurodegeneration in NDD.

Inflammation is a fundamental pathological process triggered by various factors that affect biological tissues, including injury and infection. The inflammatory response serves as the body’s primary defense against harmful substances while promoting tissue regeneration and repair (Medzhitov, 2008). However, inflammation can also lead to several negative physiological consequences. Neuroinflammation, specifically, results from factors such as toxins, infections, central nervous system (CNS) injury, or autoimmunity. Similar to other forms of inflammation, neuroinflammation can be classified as either acute or chronic, depending on the progression of the condition (Fairless et al., 2021). Acute neuroinflammation is a temporary response caused by trauma, infections, stroke, or toxins (Kaur et al., 2020), whereas chronic neuroinflammation is characterized by prolonged inflammation and is a common feature of NDD such as PD, AD, ALS, and MS. Neuroinflammation involves immunological responses within the CNS, including the activation of neurons, astrocytes, and microglia (O’Callaghan et al., 2008). Additionally, CNS inflammation may be linked to the infiltration of peripheral immune cells, which can exacerbate neuroinflammation in NDD (Li et al., 2024a). The relationship between peripheral and central inflammatory factors, as well as the potential for peripheral inflammatory factors to penetrate the CNS and contribute to neuroinflammation, remains inadequately explored. While neuroinflammation encompasses various defense processes aimed at repairing damaged glial and neuronal cells, it also serves to protect the CNS from harm and cell death. However, maintaining a balance among neuroinflammatory responses is critical; prolonged or excessive inflammatory reactions can have detrimental effects, exacerbating the progression of NDD and leading to neuronal damage or dysfunction (Kong et al., 2023). A recent study on the immunological mechanisms underlying NDD demonstrated that immune cells, such as microglia, astrocytes, T lymphocytes, B lymphocytes, and natural killer (NK) cells, are significantly associated with these disorders (Huang et al., 2022a). T lymphocyte precursors migrate to the thymus, where they differentiate into two subpopulations, CD4^+^ and CD8^+^ T cells, in response to specific surface markers (Chen and Zhu, 2013). CD4^+^ T cells are further categorized into various subgroups based on their cytokine production, transcription factor expression, and functional outcomes. This review focuses on the roles of T-cell-mediated responses in NDD. Specifically, we will explore the mechanisms by which T lymphocytes mediate neuroinflammation in NDD, uncover their involvement in the neuroinflammatory environment, and propose strategies for treating NDD by targeting T lymphocytes.

The novelty of this review lies in its perspective of T cells as a dual regulatory hub for neuroinflammation, mediating both neurodegeneration and neural repair or regeneration in the context of disease development. This work offers a theoretical foundation for precision treatments that target T cells and presents a fresh viewpoint on the regulation of the immunological environment in the field of nerve regeneration and repair. Additionally, we identify some remaining unanswered questions and limitations with the hope of providing guidance for future developments in this area.

## Search Strategy

The research cited in this review article was collected from the National Library of Medicine (PubMed) database. We searched for articles published from the inception of the database until December 31, 2024. We prioritized high-impact journals from the last 5 years while also retaining significant early studies published between 1990 and 2017. The search keywords utilized included microglia, astrocytes, T lymphocytes, B lymphocytes, NK cells, CD4^+^ T cells, CD8^+^ T cells, Th17 cells, Th1 cells, regulatory T (Treg) cells, neurodegenerative diseases, Parkinson’s disease, Alzheimer’s disease, amyotrophic lateral sclerosis, multiple sclerosis, and neuroinflammation. The search string used was as follows: (“Microglia” OR “astrocytes” OR “T lymphocytes” OR “B lymphocytes” OR “NK cells” OR “CD4^+^ T cells” OR “CD8^+^ T cells” OR “Th17 cells” OR “Th1 cells” OR “Treg cells”) AND (“neurodegenerative diseases” OR “Parkinson’s disease” OR “Alzheimer’s disease” OR “amyotrophic lateral sclerosis” OR “multiple sclerosis” OR “neuroinflammation”). The results were further screened by title and abstract, with a focus on the role of T cells in neuroinflammation related to NDD. We excluded non-English literature, conference abstracts, case reports, duplicate publications, and studies with incomplete data, ultimately including 419 references in this review. The literature retrieval process was conducted collaboratively by the research team. Professor JY oversaw the overall planning, KL was responsible for the specific retrieval operations, and other team members cross-checked the completeness and accuracy of the retrieval results.

## Activation and Differentiation of T Cells

The immune system comprises several different types of immune cells, the most common of which are lymphocytes and myeloid cells. Lymphocytes consist of T cells, B cells, and NK cells. T cells, in particular, play crucial roles in cellular immunity and the regulation and defense of the immune response. B cells contribute to humoral immunity by producing antibodies (Tangye et al., 2023), while NK cells are important innate immune cells that can eliminate tumor or virus-infected cells without prior sensitization. Myeloid cells, including neutrophils, eosinophils, basophils, dendritic cells, and monocyte-macrophages, also play significant roles in the immune system. Other cell types, such as mast cells, endothelial cells, and platelets, contribute to immune function. T cells are among the most important components of the immune system, as they coordinate various aspects of the immune response and ensure that the body’s immunological activity is precise and efficient. Based on their functions, T cells are classified into Tregs, cytotoxic T (Tc) cells, and helper T (Th) cells. Th cells support other immune cells by secreting cytokines but do not directly kill target cells. In contrast, Tc cells can specifically target and kill infected or malignant cells. Tregs primarily function to suppress the immune response and prevent its excessive activation. T cells are further classified as CD4^+^ T cells and CD8^+^ T cells based on their surface markers: CD8^+^ T cells are primarily Tc cells, while CD4^+^ T cells are mainly Th cells (Braun, 2021).

In the bone marrow, hematopoietic stem cells give rise to T lymphocytes. These stem cells differentiate into lymphoid progenitor cells, which then migrate to the thymus, a specialized immune organ. Within the thymus, these progenitor cells undergo a rigorous and organized developmental process, transforming from early pre-T cells into functionally distinct T cells (Braun, 2021). During this maturation process, T cells develop into double-negative (DN; CD4^−^CD8^−^), double-positive (CD4^+^CD8^+^), or single-positive (CD4^+^CD8^−^ or CD4^−^CD8^+^) cells. Adaptive immune cells are identified as T cells with CD4^+^ and CD8^+^ markers (Kumar et al., 2018).

### Activation and differentiation of CD4^+^ T cells

**Figure 1:**
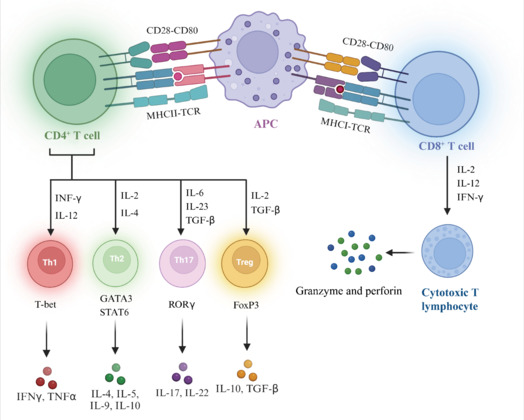
Activation and differentiation of Treg cells. CD4^+^ T cells are activated when APCs present a foreign antigen. During activation, the TCR recognizes a complex composed of MHC class II and the antigen. This activation is further enhanced by the interaction of costimulatory molecules on the surface of the APC with matching ligands on the T-cell surface. Following activation, CD4^+^ T cells differentiate into various subtypes, primarily Th1, Th2, Th17, and Treg cells, which are stimulated by different cytokines. Each of these distinct CD4^+^ T-cell subtypes has a specific function; they contribute to immune regulation and produce a variety of cytokines. After a virus invades body cells, the viral proteins are broken down within the infected cells, attached to MHC class I molecules, and then displayed on the cell surface. Moreover, CD8^+^ T cells are activated by ligands on their surface that bind to costimulatory molecules on the surface of APCs, such as CD28. Once CD8^+^ T cells are initially activated, cytokines play a crucial role in their differentiation. IL-2 and IL-12 are particularly important for the early differentiation and proliferation of CD8^+^ T cells, promoting their growth and laying the groundwork for the development of mature Tc cells. Additionally, IFN-γ can enhance the cytotoxic response. Mature cytotoxic T cells produce cytotoxic substances such as granzymes and perforin to eliminate aberrant cells, such as virus-infected and tumor cells. Created with BioRender.com. APC: Antigen-presenting cell; FoxP3: forkhead box P3; GATA3: binding protein 3; IFN-γ: interferon-gamma; IL: interleukin; MHC: major histocompatibility complex; RORγ: retinoic acid-related orphan receptor gamma; STAT5: signal transducer and activator of transcription 5; T-bet: T-box expressed in T cells; TCR: T-cell receptor; TGF-β: transforming growth factor-beta; Th1: T helper cell 1; Th17: T helper cell 17; Th2: T helper cell 2; TNF-α: tumor necrosis factor-alpha; Treg: regulatory T.

CD4^+^ T lymphocytes, also known as Th cells, derive their name from the CD4 molecules expressed on their surface. These cells are activated when antigen-presenting cells (APCs) deliver foreign antigens. To initiate their proliferation and differentiation, CD4^+^ T cells rely on three essential signals. The first signal is produced when T cell receptors recognize a complex formed by an antigen and major histocompatibility complex class II (Basu et al., 2013; **Figure 1**). The second signal occurs when a matching ligand on the surface of the T cell binds to a co-stimulatory molecule on the APC. Together, these two cues ensure T cell survival and initiate the process of multiplication. The third signal is provided by cytokines in the cell’s microenvironment, which interact with T cell receptors to guide the differentiation of CD4^+^ T cells.

Based on the cytokines they produce, CD4^+^ T cells can be classified into several subgroups, each with distinct functions (Mosmann et al., 1986). Caza and Landas (2015) summarized the subsets of CD4^+^ T cells as including Th1 cells, Th2 cells, Th9 cells, Th17 cells, Th22 cells, Th15 cells, and Tregs. In NDD, Th1 cells, Th2 cells, Th17 cells, and Tregs play significant roles in neuroinflammation. A summary of the CD4^+^ T cell development process was provided by Luckheeram et al. (2012), who stated that if the microenvironment is rich in interleukin (IL)-12 and interferon-γ (IFN-γ), the original CD4^+^ T lymphocytes tend to differentiate into Th1 cells, as guided by various signaling pathways and transcription factors. The T-box expressed in T cells is a crucial regulator of Th1 cell development and is encoded by the T-box transcription factor 21 gene. Conversely, when IL-4 is present in relatively high concentrations, it activates signal transducer and activator of transcription 6, promoting the differentiation of the initial CD4^+^ T lymphocytes into Th2 cells. In the presence of transforming growth factor-β (TGF-β), IL-6, and other cytokines, the initial CD4^+^ T lymphocytes differentiate into Th17 cells by activating relevant signaling pathways and transcription factors such as retinoic acid-related orphan receptor gamma t (RORγt). TGF-β, either alone or in conjunction with IL-2, stimulates CD4^+^ T cells to develop into Tregs. Among these subsets, Th1 and Th17 cells play essential pro-inflammatory roles, while Tregs and Th2 cells have major anti-inflammatory functions. Th1 cells enhance the ability of host cells to combat infections by producing cytokines, particularly IFN-γ. Additionally, Th1 cells can activate macrophages, improving their phagocytic and killing capabilities to help eliminate pathogens (Suzuki et al., 1988). Th2 cells, when stimulated by antigens such as extracellular microorganisms and parasites, secrete various inflammatory factors, including IL-4, IL-5, IL-9, and IL-10 (Mosmann and Coffman, 1989). Th17 cells produce IL-17, IL-22, IL-23, and other cytokines that recruit neutrophils and promote inflammatory responses at the site of infection, providing protection against extracellular bacterial and fungal infections (Shi et al., 2022). Finaly, Tregs play critical roles in maintaining immunological homeostasis and self-tolerance by producing anti-inflammatory cytokines, such as IL-10 and TGF-β, and by inhibiting the activity of various immune cells (Lee, 2018).

In summary, CD4^+^ T lymphocytes differentiate into a diverse array of subgroups (**Figure 1**). Together, they influence the body’s intricate and well-organized immune response system through their collaboration and mutual inhibition.

### Activation and differentiation of CD8^+^ T cells

CD8^+^ T lymphocytes, or Tc cells, express CD8 molecules on their surface and play a crucial role in the body’s cellular immune system by eliminating target cells. Following a virus’s invasion of a cell, viral proteins undergo several processing steps before binding to MHC class I molecules and appearing on the cell surface within an MHC complex (van Stipdonk et al., 2001). This initial step is essential for activating CD8^+^ T cells. Additionally, co-stimulatory signals are required; matching ligands on the surface of CD8^+^ T lymphocytes bind to co-stimulatory molecules, such as CD28, on the surface of APCs. When these two signals work together, they efficiently stimulate the initial CD8^+^ T cells to prepare for further differentiation.

After the initial activation of CD8^+^ T cells, various factors determine their differentiation pathways and functional maturation, with the cytokine microenvironment playing a critical role. For example, the secretion of IL-2 by Th1 cells is essential for the early differentiation and proliferation of CD8^+^ T cells, enabling them to multiply and laying the groundwork for their eventual maturation into Tc cells (Redeker et al., 2015). Furthermore, IFN-γ enhances their killing activity and guides their development to promote more effective cytotoxic responses (de Araújo-Souza et al., 2015). In certain contexts, TGF-β regulates their differentiation, preventing their excessive maturation or maintaining their functional stability based on the immunological environment (Chen, 2023a). The strength and duration of antigen stimulation also significantly influence CD8^+^ T cell differentiation. Weak or brief stimulation may lead to their incomplete differentiation and reduced killing activity, making it more challenging to eliminate target cells. In contrast, strong and prolonged stimulation promotes their rapid and complete differentiation into mature Tc cells, enhancing their killing capacity (van Stipdonk et al., 2003).

Following CD8^+^ T cells’ activation, proliferation, and differentiation, which are all regulated by distinct factors, they develop into mature Tc cells. These cells release cytotoxic substances such as granzyme and perforin. Perforin can form pores in target cell membranes, creating transmembrane channels that allow granzyme to enter the target cells. Once inside, granzyme activates apoptosis-related proteins, inducing the death of the target cells. Additionally, CD8^+^ T cells can express Fas ligands, which bind to Fas proteins on the surface of target cells. This interaction triggers the apoptosis signaling pathway within target cells, initiating cell death (Andersen et al., 2006). Together, these mechanisms ensure the efficacy and diversity of T cell responses, enabling them to respond appropriately to various immune threats. Processes involved in the activation and differentiation of CD8^+^ T cells are illustrated in **Figure 1**.

## Immune Cells Are Involved in Neuroinflammation

In a normal physiological state, immune cells in the CNS are subject to control by stringent regulatory systems that maintain a comparatively stable state. However, pathological conditions, such as brain injury, pathogen infection, and autoimmune disorders, can lead to neuroinflammation. During such events, numerous immune cells, including microglia, astrocytes, B lymphocytes, and peripheral T lymphocytes, are sequentially activated (Estes and McAllister, 2014). Following their activation, these immune cells interact in complex ways that can have various effects on neural tissues. These interactions are crucial for the onset and progression of neurological disorders, as well as for the body’s response to injury and recovery (Wyss-Coray and Mucke, 2002).

Microglia, the primary immune cells in the CNS, are usually in a resting state. However, during neurodegeneration, they utilize their pattern recognition receptors—particularly Toll-like receptors (TLRs)—to detect pathogen-associated molecular patterns and damage-associated molecular patterns, as well as signals in the form of extracellular matrix components released by injured neurons (Stephenson et al., 2018). Subsequently, microglia transition from a resting to an active state, changing from a branched morphology to an amoeba-like shape. Concurrently, the expression levels of various cytokines, chemokines, reactive oxygen species, reactive nitrogen species, and other substances are significantly upregulated, and their internal expression mechanisms are appropriately modified (García-Revilla et al., 2019). The function of activated microglia is dualistic. On one hand, they can phagocytose and eliminate pathogens and cellular debris, support nerve tissue regeneration, and play a significant role in maintaining the regular function of the nervous system. On the other hand, when microglia become over-activated, they release large quantities of pro-inflammatory cytokines, such as tumor necrosis factor-alpha (TNF-α), IL-1β, and IL-6 (Hickman et al., 2018). This excessive activation exacerbates the initial neuroinflammatory response and accelerates the progression of NDD by directly harming nerve cells.

Like microglia, astrocytes are also activated by signals of inflammation or injury within the CNS. They can recognize pathogens or injury-related signals through surface receptors, with their activation signals partially derived from cytokines such as TNF-α and IL-1β released by microglia (Liddelow et al., 2017). Following activation, astrocytes undergo structural changes, such as hyperplasia and hypertrophy, which contribute to the formation of glial scars. Although this process can help contain inflammation and protect peripheral nerve tissue, the resulting hyperplastic scar can inhibit nerve regeneration and impair functional recovery. Activated astrocytes also secrete a variety of cytokines and chemokines that interact with microglia, collectively shaping the neuroinflammatory environment and influencing pathogenic alterations associated with neuroinflammation (Colombo and Farina, 2016).

In addition to microglia and astrocytes, T lymphocytes and B lymphocytes also participate in neuroinflammation. Under normal physiological conditions, the blood–brain barrier (BBB) effectively blocks peripheral immune cells, safeguarding the CNS. However, when the BBB is compromised and becomes more permeable, peripheral immune cells can infiltrate the CNS. Different types of peripheral immune cells play distinct roles once they enter the CNS; for instance, Tc cells identify and destroy virus-infected nerve cells, as well as aberrant cells such as tumor cells (Fang et al., 2018). Th cells regulate the immune response by secreting various cytokines, stimulating macrophages, and assisting B cells in antibody production. Th cells are essential for coordinating the entire immune response in the CNS, ensuring an organized and effective reaction (Zhu and Zhu, 2020). Tregs help maintain immunological homeostasis and prevent excessive immune reactions, thus they play a critical role in moderating immune responses in the CNS to prevent further damage from an overactive immune system (Singer et al., 2024). B lymphocytes, which primarily function in humoral immunity, are also involved in neuroinflammation. Under normal conditions, B lymphocytes are typically found in peripheral lymphatic organs. However, following neurodegeneration, they can cross the BBB and enter the CNS in response to chemokines and other stimuli. Once inside the CNS, B lymphocytes differentiate into plasma cells, which produce specific antibodies that can bind to antigens on the surfaces of nerve cells, activate the complement system, and initiate various processes that may harm nerve cells (Taher et al., 2017).

The mechanism of neuroinflammation is intricate and involves multi-faceted interactions among various immune cells, followed by a series of subsequent reactions.

## Regulation of Neuroinflammation by Immune Cells: Core Role of T Cells and Their Synergistic Mechanisms in Parkinson’s Disease

Among progressive NDD, PD is the second most common condition (Dorsey and Bloem, 2018). A key characteristic of PD is a decline in motor ability due to injury in the dopaminergic nigrostriatal system (Jagadeesan et al., 2017). As the human population ages, the prevalence of PD is expected to rise significantly, with an estimated 12 million PD patients worldwide by 2040 (Dorsey and Bloem, 2018). PD is marked by a range of motor symptoms, including bradykinesia, resting tremor, rigidity, and alterations in posture and gait (Tolosa et al., 2021).

The pathogenesis of PD involves multiple pathways, including epigenetic changes, mitochondrial dysfunction, oxidative stress, abnormalities in lysosomal or vesicular transport, synaptic transport anomalies, apoptosis, autophagy, ubiquitination, and inflammatory responses (Cacabelos, 2017; Bloem et al., 2021). Currently, there is no known cure for PD, and the underlying pathophysiology remains to be fully elucidated. A notable aspect of PD is the role of the inflammatory response. Early studies identified activated microglia in the brains of PD patients (McGeer et al., 1988b; Hirsch et al., 1998). Subsequent research revealed the presence of altered cytokines and T cell infiltration in the brains of patients with PD, with increased levels of several cytokines identified in the cerebrospinal fluid (CSF) and serum (Brodacki et al., 2008). Brain slices from PD patients have also been shown to contain increased pro-inflammatory factors, microglial activation, and T lymphocyte infiltration (McGeer et al., 1988a). Neuroinflammation in PD involves a complex mechanism in which the aberrant accumulation of α-synuclein (α-syn) triggers both innate and adaptive immune responses. Neuroinflammation leads to α-syn misfolding and aggregation (Allen Reish and Standaert, 2015), ultimately resulting in the death of dopaminergic (DA) neurons in PD patients.

The neuroinflammatory response in PD is mediated by both peripheral immune cells and the CNS. Studies have shown that peripheral and central inflammation is elevated in PD (Imamura et al., 2003; Nagatsu and Sawada, 2005; Kim et al., 2022), and neuroinflammation is prevalent in the brains of most PD patients (Zhang and Gao, 2022). Moreover, patients with PD exhibit significantly higher levels of IL-1β, IL-2, IL-6, epidermal growth factor, and TGF-α in the dopaminergic striatum (Mogi et al., 1994). A meta-analysis revealed elevated peripheral levels of IL-6, TNF-α, IL-1, and IL-2 in PD patients (Qin et al., 2016).

The inflammatory response triggered by these factors is closely associated with both non-motor and motor symptoms in patients with PD. For instance, studies have reported that higher levels of TNF-α in the peripheral blood correlate significantly with the severity of PD symptoms, including cognitive difficulties, depression, and sleep disturbances (Menza et al., 2010; Lian et al., 2024). Additionally, both blood and CSF from PD patients demonstrate a substantial increase in activated T cells (Schröder et al., 2018). The aggregation of α-syn is a hallmark of PD, and the injection of α-syn into the striatum of mice was found to result in an increase in T lymphocytes in both the peripheral blood and the CNS (Earls et al., 2019).

### Crucial contribution of microglia and their interaction with T cells to neuroinflammation associated with Parkinson’s disease

Microglial cells, which account for 5%–12% of CNS cells, exhibit varying densities across different brain regions (Lawson et al., 1990). These cells are considered the macrophages of the CNS. In a healthy brain, microglia communicate with neurons and astrocytes to detect changes in the surrounding environment (Allen Reish and Standaert, 2015), and when the CNS is injured by pathogenic agents, microglia become activated (Djukic et al., 2006). Activated microglia can be categorized into pro-inflammatory M1 and anti-inflammatory M2 variants. Overactive M1 microglia produce pro-inflammatory mediators that can damage neurons and contribute to the progression of PD (Allen Reish and Standaert, 2015). In contrast, M2 microglia secrete neuroprotective factors and work against the development of PD (Jha et al., 2016).

In 1988, the first neuropathological evidence was published indicating an association between microglial cells and PD (McGeer et al., 1988a). The substantia nigra (SN), putamen, hippocampus, and cortex of PD patients all contain activated microglia (Imamura et al., 2003), and imaging studies have revealed that a key characteristic of the brain in PD is the proliferation of microglia (Ouchi et al., 2005; Gerhard et al., 2006). Peripheral adaptive immunity may play an indirect role in the activation of microglia in PD (Liu et al., 2022). Activated microglia simultaneously release neurotrophic and neurotoxic factors in NDD, and while these factors may promote neuronal survival, they can also be detrimental to neurons (Colonna and Butovsky, 2017). Microglia produce various cytokines in the CNS, and upon activation, they release pro-inflammatory cytokines and increase their MHC-II expression (Imamura et al., 2003). In PD, cytokine release originates from ATP-induced microglial chemotaxis and α-synuclein-triggered NADPH oxidase activation, with both pathways synergistically amplifying neuroinflammatory responses to DA injury (Davalos et al., 2005; Zhang et al., 2005).

**Figure 2:**
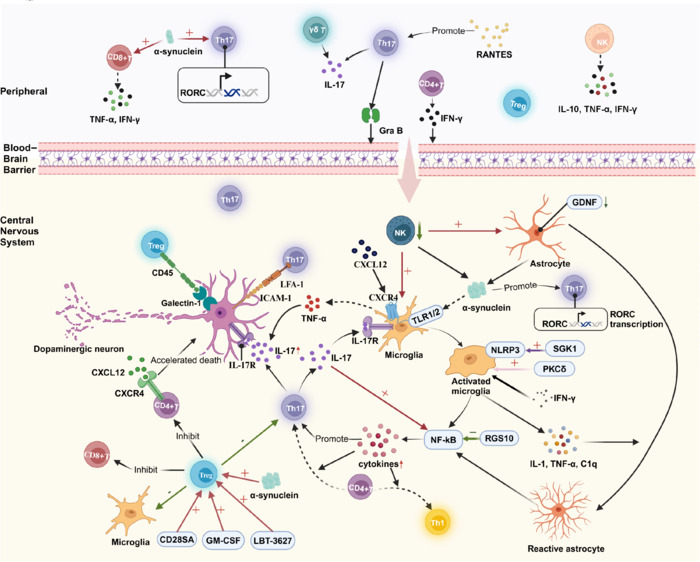
Role of neuroinflammation caused by CD4^+^ T cells in Parkinson’s disease. CD4^+^ T cells infiltrate the peripheral blood of individuals with PD. IFN-γ secreted by CD4^+^ T cells and granzyme B expressed by Th17 cells can weaken endothelial tight junctions, promoting immune cell infiltration into the CNS. CD4^+^ T cells are categorized into proinflammatory Th17 and Th1 cells, as well as anti-inflammatory Tregs. In the CNS of patients with PD, IL-17 secreted by Th17 cells leads to the overexpression of IL-17R and the activation of NF-κB, resulting in the secretion of proinflammatory cytokines and the promotion of neuroinflammation. LFA-1 expressed by Th17 cells interacts with the LFA-1 receptor ICAM-1 on DA neurons, causing direct damage to these neurons by Th17 cells. Additionally, TNF-α mediates the synergistic damage to DA neurons induced by IL-17A. In contrast, Tregs play a protective role in DA neurons. The interaction between CD45 on the surface of Tregs and galectin-1 on the surface of DA neurons mediates a neuroprotective effect. Unfortunately, the number of Tregs tends to decrease in PD patients. Moreover, cytokines also mediate the activation of microglia and astrocytes. Activated microglia indirectly promote the differentiation of CD4^+^ T cells into proinflammatory Th1 and Th17 subtypes, exacerbating neuroinflammation in PD. Ultimately, increased neuroinflammation leads to greater accumulation of α-syn and the death of dopaminergic neurons. Created with BioRender.com. CNS: Central nervous system; CXCL12: C–X–C chemokine ligand 12; CXCR4: C–X–C chemokine receptor type 4; GDNF: glial cell line-derived neurotrophic factor; GM-CSF: granulocyte-macrophage colony-stimulating factor; Gra B: granzyme B; ICAM-1: intercellular adhesion molecule 1; IFN-γ: interferon-gamma; IL: interleukin; IL-17R: interleukin-17 receptor; LFA-1: leukocyte function-associated antigen 1; NK: natural killer cell; NLRP3: nucleotide-binding oligomerization domain, leucine-rich repeat and pyrin domain-containing protein 3; NF-κB: nuclear factor kappa-B; PKCδ: protein kinase C delta; RANTES: regulated on activation, normal T-cell expressed and secreted; RGS10: regulator of G-protein signaling 10; RORC: retinoic acid receptor-related orphan receptor C; SGK1: serum/glucocorticoid related kinase 1; Th1: T helper cell 1; Th17: T helper cell 17; Th2: T helper cell 2; TLR 1/2: Toll-like receptor 1/2; TNF-α: tumor necrosis factor-alpha; Treg: regulatory T cell.

Numerous studies have demonstrated that the neuroinflammation induced by microglia is closely linked to α-syn (Croisier et al., 2005; Su et al., 2008). Microglia promote neuronal inflammation in response to the misfolding and accumulation of α-syn (He et al., 2020). In PD, microglial cells engulf dead cells and assist in clearing α-syn (Béraud et al., 2013). Notably, α-syn acts as an endogenous agonist of TLR2, which activates microglia and renders them neurotoxic (Daniele et al., 2015; **Figure 2**). The overexpression of α-syn activates microglia, leading to the release of pro-inflammatory cytokines (Kim et al., 2013). Therefore, the interaction between α-syn and microglia is crucial to the pathophysiology of PD.

Despite being the brain’s first line of defense, microglia can also produce neurotoxic substances that contribute to neuronal damage. Microglial activity has been linked to neuronal loss in PD (Ouchi et al., 2005; Kuter et al., 2018), as in a study that reported that the α-syn-induced loss of DA neurons was mediated by microglial activation (Subbarayan et al., 2020). The activation of the NOD-like receptor protein 3 (NLRP3) inflammasome in microglia is a crucial factor in the 1-methyl-4-phenyl-1,2,3,6-tetrahydropyridine (MPTP)-induced PD mouse model (Lee et al., 2019). Kwon et al. (2021) demonstrated that inhibiting microglial activation blocks NLRP3 and nuclear factor kappa B (NF-κB) inflammasomes, thereby preventing the degeneration of dopamine neurons (**Figure 2**). Another study showed that regulator of G-protein signaling 10 had a neuroprotective effect in a PD rat model and reduced NF-κB activation in activated microglia (Lee et al., 2011; **Figure 2**). Additionally, microglia regulate the release of cytokines, which contribute to the death of DA neurons, and these neurons are adversely affected by microglial-derived TNF-α (De Lella Ezcurra et al., 2010; Wenker et al., 2023). One potential strategy to protect these neurons is to suppress TNF-α expression. Microglial activation can trigger cytokine storms via IFN-γ, further increasing damage to DA neurons (Chien et al., 2016). Moreover, protein kinase C delta (PKCδ) plays a crucial role in regulating inflammatory cytokines in microglia (Hayden and Ghosh, 2004), and the knockout of PKCδ in mice treated with MPTP and lipopolysaccharide prevented the death of DA neurons (Gordon et al., 2016; **Figure 2**). Consequently, we know that microglial activation has a deleterious impact on PD, and targeting this process may provide a treatment strategy to prevent DA cell death.

M2 microglia promote the release of anti-inflammatory mediators and protect neurons from the damage associated with PD. Therefore, a potential treatment method for PD involves inducing the transition of M1 microglia towards the M2 phenotype. The Kir6.1/K-ATP channel, which links cellular metabolism and membrane potential, can convert microglia into a protective M2 phenotype, thereby mitigating DA neuron death (Du et al., 2018). Transplanting human menstrual blood-derived endometrial stem cells was used to regulate microglia, reducing movement abnormalities and neuronal damage in 6-hydroxydopamine-induced PD rats (Li et al., 2023a). The stimulation of T lymphocytes by microglia is also a crucial step in PD. Activated microglia was shown to facilitate the infiltration of Th17 cells into the brain, enhancing their pro-inflammatory influence in PD mouse models (Liu et al., 2019b).

### Crucial contribution of astrocytes and their interaction with T cells to the neuroinflammation associated with Parkinson’s disease

Astrocytes, another subtype of glial cells, are widely distributed throughout the brain and spinal cord, where they play a crucial role in maintaining CNS homeostasis. Like microglia, astrocytes contribute to neuroinflammation and can be categorized into two types: A1 and A2. A1 astrocytes induce the release of pro-inflammatory cytokines, which can have toxic effects on neurons in PD (Liddelow and Barres, 2017). In contrast, A2 astrocytes protect neurons by upregulating neurotrophic proteins, including prokineticin-2, chitinase-like 3, frizzled class receptor 1, and arginase 1 (Liddelow and Barres, 2017; Neal et al., 2018). Following brain injury from conditions such as infection, inflammation, trauma, and NDD, astrocytes become reactive astrocytes (Barreto et al., 2007; Hamby and Sofroniew, 2010; Xiong et al., 2011). Additionally, microglia can induce the formation of neurotoxic reactive astrocytes by secreting TNF-α and IL-1α (Liddelow et al., 2017).

Astrocytes also mediate neuroinflammation in PD through α-syn (Braak et al., 2007). Under pathological conditions, astrocytes aggregate α-syn and secrete various cytokines and chemokines, and the α-syn found in astrocytes is believed to originate from neurons (Lee et al., 2010). The α-syn that accumulates in neurons is endocytosed by astrocytes; however, this α-syn accumulation leads to mitochondrial dysfunction in astrocytes, resulting in an inflammatory response (Lindström et al., 2017).

In the SN of both PD patients and MPTP-induced monkey models, reactive astrocytes have been reported to be prevalent and primarily localized to these regions (Miklossy et al., 2006). Thus, the loss of DA neurons in PD is facilitated by the activation of reactive astrocytes. Glial cell line-derived neurotrophic factor (GDNF) promotes dopamine uptake and neuron survival (Lin et al., 1993). GDNF levels increase early in PD as a potential neuronal defense mechanism (Werner et al., 2008); however, as PD progresses, GDNF levels decrease (Rangasamy et al., 2010). In MPTP-induced PD animal models, researchers have found the astrocyte-mediated activation of NF-κB increases the production of pro-inflammatory cytokines and apoptosis-related proteins, resulting in the death of DA neurons (Aoki et al., 2009). Inhibiting astrocyte activation also reduces the levels of pro-inflammatory factors, thereby preventing the further degeneration of DA neurons (Yang et al., 2009). In rat PD models, prolonged astrocyte dysfunction accelerates the degeneration of DA neurons (Kuter et al., 2018).

Neuroinflammation is significantly influenced by the interaction between microglia and astrocytes. Liddelow et al. (2017) showed that the secretion of IL-1, TNF-α, and C1q by reactive microglia leads to the formation of reactive A1 astrocytes, ultimately causing neuronal damage (Liddelow et al., 2017). Conversely, astrocytes also modulate microglial activation and the neuroinflammatory response induced by microglia (Jo et al., 2017). Additionally, the ability of astrocytes to act as APCs in response to α-syn fibrils makes them essential for the activation of T cells.

In PD, the cytokines IL-17A and TNF-α secreted by CD4^+^ T cells can significantly enhance the responses of astrocytes (MacMahon Copas et al., 2024). Additionally, astrocytes in PD patients have the capacity to activate T cells and function as potential APCs, and astrocytes expressing MHC-II surround infiltrating CD4^+^ T cells in the brain and around blood vessels. Furthermore, astrocytes that have accumulated α-syn express the necessary signals to activate invading T cells (Rostami et al., 2020). Astragaloside IV, extracted from the traditional Chinese medicinal herb Astragalus membranaceus, may effectively prevent aberrant interactions between astrocytes and CD4^+^ T cells. In a rotenone-induced PD rat model, the extract interfered with the abnormal colocalization of MHC-II and α-syn in astrocytes by inhibiting the overactivation of α-syn-specific CD4^+^ T cells (Wang et al., 2024a).

### Dual regulatory role of natural killer cells in the neuroinflammation of Parkinson’s disease

NK cells, which play a key role in innate immunity, are granular lymphocytes that release a range of proinflammatory cytokines, including IL-10, TNF-α, and IFN-γ. The number and function of NK cells are increased in PD (Niwa et al., 2012; Cen et al., 2017; Jiang et al., 2017; Sun et al., 2019; Holbrook et al., 2023). NK cells become activated in the early stages of PD (Holbrook et al., 2023), and there is an increase in the level of resting NK cells in the blood of PD patients (Huang et al., 2021). The deliberate depletion of NK cells in PD mice led to an increase in the number of microglia and astrocytes invading the SN, which was associated with heightened neuroinflammation and worsened motor impairments (Earls et al., 2020). Furthermore, elevated NK cell cytotoxicity was seen to contribute to DA neuronal injury in a PD rat model (Grembecka et al., 2021). NK cells migrate from the periphery to the PD brain, where they target and kill malfunctioning DA neurons, a process facilitated by BBB disruption.

NK cells additionally serve a defense role in PD. Infiltrated NK cells co-localize with α-syn and DA neurons (Guan et al., 2022) and have been shown to clear accumulated α-syn *in vivo* (Earls et al., 2020). Thus, NK cells may play a protective role in the disease. However, further research is needed to understand the alterations in NK cells and their subpopulations, as well as their specific involvement in PD.

An *in vivo* animal investigation revealed that NK cells are a crucial source of early IFN-γ, which is necessary for the polarization of T cells into Th1 cells in lymph nodes. The priming and polarization of Th1 cells depend on the recruitment of NK cells and the release of IFN-γ (Martín-Fontecha et al., 2004). T cells also have the capacity to regulate NK cells; for instance, T cell-secreted IL-2 is known to activate CD56^bright^ NK cells (Fehniger et al., 2003). This highlights the dynamic interactions occurring between innate and adaptive immune cells and underscores the importance of studying these interactions to gain a comprehensive understanding of the immune response process.

### Crucial contribution of B cells and their interaction with T cells to the neuroinflammation associated with Parkinson’s disease

B lymphocytes are bone-marrow-derived pluripotent stem cells that play a crucial role in acquired immunity. When stimulated by an antigen, B cells can differentiate into plasma cells, which produce and secrete antibodies and thereby primarily mediate humoral immunity. In the brains of patients with PD, the majority of antibodies are directed against α-syn. A study gathered evidence of a substantial increase in anti-α-syn IgG2 and IgG4 antibodies in PD, while finding that anti-α-syn IgM levels were significantly decreased (Folke et al., 2019). Moreover, a negative correlation between IgM levels and the progression of the disease has been found, and an increase in anti-α-syn antibodies may contribute to the progression of PD. For instance, injecting IgG into the SN of rats led to a significant increase in vascular inflammation, microglial infiltration, and the destruction of DA neurons (Chen et al., 1998). Additionally, IgG has been shown to enhance the DA neuronal death induced by microglia *in vitro* (He et al., 2002).

Some researchers have suggested that B cells may have a protective role in PD, and studies have shown that there are fewer B lymphocytes in the peripheral blood of both PD mice and patients than healthy individuals (Stevens et al., 2012; Idova et al., 2021; Scott et al., 2023). In terms of the B cell subtypes involved in PD, the number of blood B cells that produce pro-inflammatory cytokines increase, while regulatory B cells and total B cell counts decrease (Li et al., 2022). Furthermore, B cell-related genes, such as *CD19*, *CD22*, and *CD79A*, were found to be decreased in PD patients (Kobo et al., 2016). Additionally, PD cases have an increase in memory B cell clones and a decrease in naive B cell clones, suggesting that B cell activation is elevated (Wang et al., 2022b).

The reduction in peripheral blood T follicular helper (Tfh) cells in PD patients is associated with a decline in B cell populations (Li et al., 2022). In addition to producing antibodies, B cells contribute to neuroinflammation in PD through antibody-independent mechanisms. These mechanisms can involve the cells acting as APCs or triggering T cell activation by releasing cytokines. For instance, Th1 and Th17 cell responses were found to be decreased in B cell-specific MHC-deficient mice (Molnarfi et al., 2013). However, further research is needed to elucidate the pathogenic role of B cells in PD and to promote the development of novel therapeutic concepts.

### Multifaceted roles of T cells in the neuroinflammation and pathophysiology of Parkinson’s disease

T lymphocytes are bone marrow-derived lymphoid stem cells that undergo differentiation and maturation in the thymus. Once mature, T cells are transported via the blood and lymphatic systems to various tissues and immune organs throughout the body to perform their immunological functions. The brain was once thought to be an immune-privileged organ, where immune cells were unable to cross the BBB. However, a healthy brain contains a small number of beneficial T cells that help maintain normal brain function and promote tissue regeneration in various neurological conditions (Smolders et al., 2018; Evans et al., 2019). CD4^+^ T cells play a particularly important role in the healthy brain. Studies have shown that there are very few CD4^+^ T cells in the brains of healthy humans and mice, and those present are typically located near microglia (Pasciuto et al., 2020). When these CD4^+^ T cells are lost, microglial behavior becomes abnormal, leading to various brain dysfunctions (Pasciuto et al., 2020). In contrast, during disease states, T cells can become harmful. PD patients exhibit abnormal humoral and cellular immune responses in their peripheral blood. Kortekaas et al. (2005) first proposed that BBB disruption is a key aspect of PD pathophysiology. When the BBB is compromised, the brain becomes more accessible to a range of peripheral inflammatory cells and substances, resulting in neuroinflammation and potentially leading to neuronal death.

T cell activation is associated with a variety of conditions, including autoimmune diseases, tumors, infections, and NDD. Numerous studies have highlighted the close relationship between T cells and the onset or progression of NDD, particularly PD. The crucial role of T cells in PD is garnering increasing attention. In MPTP-treated mice, T cell infiltration has been observed in lesions of the nigrostriatal circuit; however, the T cells did not proliferate in uninjured areas of the brain. This indicates that T cell infiltration is a subsequent and tightly regulated pathogenic process associated with neuronal cell death in PD (Brochard et al., 2008). Furthermore, CD4^+^ and CD8^+^ T cells, but not B cells, have been detected in the SN pars compacta region of patients with PD, suggesting that T cells play a significant role in the disease (Brochard et al., 2008).

#### Summary of T cell Function in Parkinson’s disease

The presence of T cells in the brains of PD patients is now a universally recognized sign. γδ T cells are unconventional CD3^+^ T cells found in the blood and tissues, primarily comprising CD4^−^ CD8^−^ T cells, which play roles in mediating signals between the innate and adaptive immune systems (Dar et al., 2014). PD patients exhibit a higher percentage of γδ T cells in both their blood and CSF than patients with other neurological diseases (Fiszer et al., 1994). Additionally, the peripheral blood of PD patients shows an increase in γδ T cells that produce IL-17, indicating they have an activated immunological response to the disease (Diener et al., 2023). The proportion of T lymphocytes among all lymphocytes is reflected in the CD3^+^ T cell subgroup. McGeer et al. (1988a) first identified the CD3^+^ T cell marker in the brains of PD patients in 1988, and subsequent studies have shown that both the proportion of activated T cells and the total T lymphocyte count in the CSF of PD patients are elevated (Schröder et al., 2018). Chen et al. (2021b) further demonstrated that PD patients had increased peripheral immunological activation and a higher number of CD3^+^ T cells than healthy controls. The infiltration of T cells into the brains of PD patients has also been evidenced in rodent studies (Kurkowska-Jastrzebska et al., 1999; Williams et al., 2021). Notably, CD3^+^ T cell levels in the peripheral blood were significantly elevated in both young and aged transgenic A53T α-syn PD mice (Idova et al., 2021).

Conversely, other studies have reported that PD patients with peripheral immunological alterations exhibit a reduced percentage of T lymphocytes (CD3^+^ cells), particularly activated T lymphocytes (CD4^+^CD25^+^ cells) (Cen et al., 2017; Rocha et al., 2017; Sun et al., 2019). A meta-analysis involving 943 PD patients demonstrated a decrease in CD3^+^ T cells in their peripheral blood (Jiang et al., 2017). Moreover, PD patients have a greater number of activated lymphocytes, apoptosis-prone lymphocytes, and central memory T cells than healthy individuals; however, these levels significantly decline as the disease progresses (Garfias et al., 2022). A study indicated there is a correlation between the severity of PD and the overall decline in CD3^+^ T cells in the blood, as well as the expression of inflammatory genes within T cells (Bhatia et al., 2021). Additionally, the number of CD3^+^ T cell subsets in the peripheral blood is associated with the cognitive level of patients with PD. Specifically, PD patients who have higher proportions of CD3^+^ T cells exhibit slower rates of cognitive decline (Xiao et al., 2023).

The abnormal aggregation of α-syn may be closely associated with T cell infiltration in PD (Iba et al., 2020). A previous case-control study provided evidence that the α-syn-specific T cell response is a hallmark of the preclinical and early stages of PD (Mollenhauer et al., 2011), with these cells present prior to the onset of motor symptoms and the clinical diagnosis of PD (Lindestam Arlehamn et al., 2020). Following the onset of motor symptoms, T cell responses to α-syn diminish with increasing disease duration (Lindestam Arlehamn et al., 2020). Peptides derived from α-syn have been shown to induce cytotoxic and Th cell responses in PD patients (Sulzer et al., 2017). Williams et al. (2023) proposed that the inflammatory T cell responses in PD may target autoantigens, such as α-syn, more specifically than typical foreign antigens. These T cells may be autoreactive, identifying misprocessed α-syn as a foreign entity and triggering an autoimmune reaction (Garretti et al., 2019). Furthermore, the non-motor symptoms of PD are characterized by the infiltration of autoreactive T cells induced by α-syn. Compared to healthy individuals, both PD patients without dementia and those with dementia exhibit significantly higher levels of infiltrating T lymphocytes in the SN (Kouli et al., 2020). In PD patients with dementia, various brain regions show heightened immunological responses, including increased α-syn pathology, microglial activation, astrocyte activation, and elevated levels of pro-inflammatory cytokines (Kouli et al., 2020). In an investigation of AAV-A53T-α-syn PD mice, a pathogenic α-syn-specific T cell response was implicated in their DA neurodegeneration (Karikari et al., 2022). The processes of α-syn deposition and T cell infiltration may reinforce each other. A recent study indicated that α-syn aggregation and neuronal death occur prior to the invasion of cytotoxic CD8^+^ T lymphocytes in the SN of PD models (Gonzalez-Latapi et al., 2022). This suggests that CD8^+^ T cells may contribute to α-syn aggregation and neuronal death in PD, while similar findings have not yet been reported for other T cell subsets. Thus, further studies are warranted to explore the dynamic progression of T cell responses in PD and to investigate whether targeting T cells for immunotherapy could be applied to modify the neuroinflammation associated with the disease.

Taken together, these findings suggest that changes in T lymphocyte populations may be contributing factors in PD. Further studies involving larger cohorts of PD patients are necessary, and future research should aim to correlate T cell alterations with disease severity and other clinical factors to identify the most relevant gene targets for expression analysis. This approach would complement the T cell count data and enhance the specificity of disease severity tracking methods.

#### CD8^+^ T cells in Parkinson’s disease

Numerous publications have addressed the alterations seen in CD8^+^ T cells in PD. PD patients exhibit an increased number of CD8^+^ T cells, and this elevation is positively associated with neuronal death (Galiano-Landeira et al., 2020). Notably, α-syn aggregation and neuronal death occur prior to CD8^+^ T cell infiltration in the SN (Galiano-Landeira et al., 2020). However, a decrease in CD8^+^ T cells in the SN was reported in Pink1/Parkin double-knockout PD rats (Lamberty et al., 2023). The high expression of BBB-permeable molecules may play a significant role in facilitating CD8^+^ T cell infiltration into the CNS of patients with PD.

Some controversy exists regarding the alterations that occur in CD8^+^ T cells in the peripheral circulation of patients with PD. Bhatia et al. (2021) proposed that CD8^+^ T cell depletion is the primary mechanism underlying the decrease in T lymphocyte levels in the blood of patients with PD, and this reduction correlates with the severity of the condition. Studies categorizing CD8^+^ T cells have indicated there is a reduction in early CD8^+^ T cell counts in the blood of PD patients (He et al., 2022). In contrast, single-cell sequencing of the peripheral blood of PD patients revealed a significant increase in terminal effector CD8+ T cell clones (Wang et al., 2021). Additionally, patients with early-to-middle-stage PD have been found to have higher blood levels of CD45RA^+^ effector memory CD8^+^ T cells (Capelle et al., 2023), which secrete elevated levels of TNF-α and IFN-γ cytokines, resulting in increased cytotoxicity (Hamann et al., 1997).

#### Important role of CD4^+^ T cells in Parkinson’s disease

Another significant component of T lymphocytes—the frequency of CD4^+^ T cells—fluctuates in the peripheral blood of PD patients (Bas et al., 2001). Williams et al. (2021) reported that α-syn-responsive T cells in the SN are primarily composed of IFN-γ-producing CD4^+^ T cells, as the neurodegenerative effects of α-syn are diminished following the knockout or pharmacological depletion of CD4^+^ T cells, but not CD8^+^ T cells, in PD mice. Therefore, CD4^+^ T cells play a crucial role in the etiology of PD.

Conflicting results have been reported regarding the outcomes of CD4^+^ T cells in PD. Some studies have indicated that peripheral circulating CD4^+^ T cells are decreased in PD patients (Stevens et al., 2012; Chen et al., 2015a; Kustrimovic et al., 2018; Li et al., 2023c). Furthermore, PD patients exhibit significantly lower levels of both CD4^+^ and CD8^+^ T cells in their peripheral blood (Wang et al., 2021). A hallmark of autoimmune disorders or immunological deficits is an imbalance between CD4^+^ T cells and CD8^+^ T cells, which may contribute to immune system dysfunction in patients with PD. A meta-analysis also reported a reduction in CD4^+^ T lymphocytes in the blood of individuals with PD (Jiang et al., 2017). Conversely, one study found that PD patients have a higher number of CD4^+^ T lymphocytes than healthy controls, resulting in a greater CD4^+^ T cell/CD8^+^ T cell ratio in PD patients (Chen et al., 2021b). Additionally, CD4^+^ T cells from PD patients exhibit aberrant functions, including reduced phenotypic migration ability and diminished intracellular mitochondrial function, compared to those from healthy individuals (Mamula et al., 2022). These changes may contribute to immune system dysfunction and increased infection rates in PD patients (Mamula et al., 2022). Further research has demonstrated that PD patients have decreased naive CD8^+^ T cells and an increase in late-differentiated CD4^+^ T cells compared to healthy controls (He et al., 2022).

The early stages of T cell differentiation are characterized by their higher proliferation capacity and lower cytotoxic effects, while an increase in late-differentiated T cells is indicative of immune aging. Consequently, PD is closely associated with the aging of the adaptive immune system (He et al., 2022). However, other studies have reported no significant differences in CD4^+^ T cells between PD patients and healthy controls (Cen et al., 2017; Schröder et al., 2018). Therefore, further research on the number of CD4^+^ T cells in PD patients is necessary. Importantly, these studies should take into account factors such as the age, gender, symptoms, and other characteristics of patients with PD.

Most studies examining alterations in CD4^+^ T lymphocytes in the brains of PD patients have been conducted using animal models (Brochard et al., 2008; Williams et al., 2021). For instance, the injection of preformed fibrillary α-syn into the mouse striatum resulted in a significant increase in CD4^+^ T cell infiltration in the CNS, but not in the blood (Earls et al., 2019). One possible explanation for the CNS infiltration of CD4^+^ T cells is that the IFN-γ secreted by these cells weakens the tight junctions of endothelial cells, thereby facilitating their infiltration (Yan et al., 2023; **Figure 2**). Both peripheral and central immune disorders related to CD4^+^ T cells have been observed in PD patients and animal models. However, there are insufficient data to establish a substantial association between immune cell alterations in the central and peripheral regions.

DA can effectively regulate inflammation via two main subtypes of DA receptors: D1-like and D2-like receptors. D1-like receptors include DRD1 and DRD5, while D2-like receptors comprise DRD2, DRD3, and DRD4. Studies have shown that DRD1 and DRD2 primarily inhibit inflammation (Shao et al., 2013; Yan et al., 2015), whereas DRD3 and DRD5 tend to promote inflammation (Franz et al., 2015). Among the latter, DRD3 plays a crucial role in regulating CD4^+^ T cells (Contreras et al., 2016). When human-activated CD4^+^ T cells are stimulated *in vitro* with specific DRD3 agonists, there is an increase in IFN-γ and a decrease in IL-10 (Ilani et al., 2004). In a PD mouse model, abnormalities in the DRD3 protein expressed on CD4^+^ T cells were seen to reduce neuroinflammation and neurodegeneration, suggesting that the DRD3 expressed on CD4^+^ T cells facilitates disease progression (González et al., 2013). The treatment of CD4^+^ T cells in MPTP mice with DRD3 antagonists markedly reduced the animals’ neurodegeneration and mobility abnormalities (Elgueta et al., 2019). Moreover, inhibiting DRD3 before the onset of clinical symptoms may affect the function of CD4^+^ T cells and hinder the progression of PD. However, Nagai et al. (1996) reported that the level of DRD3 mRNA is reduced in patients with PD, with the degree of downregulation correlating with the stage of the disease. A recent study found that CD4^+^ T cells exhibit lower DRD3 expression levels than other cell types (Kim et al., 2018). Due to conflicting evidence regarding the significance of CD4^+^ T cells and DRD3 in the etiology of PD, further research is needed to fully elucidate the relevant interactions. Additionally, the function of DRD2 on CD4^+^ T cells should not be overlooked. In a PD mouse model, the specific knockout of Drd2 in CD4^+^ T cells resulted in increased dopamine neurotoxicity, motor impairment, microglial activation, and the polarization of CD4^+^ T cells toward the Th1 and Th17 phenotypes (Liu et al., 2021).

#### Th17 cells are major proinflammatory CD4^+^ T cells in Parkinson’s disease

In 2005, a new subtype of CD4^+^ Th cell, known as Th17 cells, was discovered (Wynn, 2005) that express the RORγt transcription factor on their surface (Ivanov et al., 2006). The RORC gene, which produces RORγt, regulates the development of Th17 cells (Ivanov et al., 2006). Th17 cells are associated with autoimmune and inflammatory neurological diseases, including PD, MS, AD, and schizophrenia (Tahmasebinia and Pourgholaminejad, 2017). Furthermore, certain differentially expressed genes in PD patients verses healthy controls have been implicated in the signaling pathways that govern Th17 cell development (Chen et al., 2021a).

There have been contradictory results regarding the frequency of Th17 cells in patients with PD. Several studies have reported a consistent increase in the number of IL-17-producing cells among PD patients (Yang et al., 2017; Storelli et al., 2019; Liu et al., 2022). Specifically, Th17 cells were significantly elevated in the peripheral blood of PD patients compared to that of healthy controls (Chen et al., 2017), and PD patients exhibited higher proportions of Th1 and Th17 cells than Th2 cells and Tregs (Chen et al., 2015a). Diener et al. (2023) reported elevated levels of IL-17 secreted by γδ T cells in patients with PD; however, the contribution of γδ T cells to PD has not been extensively documented. Rocha et al. (2017) found lower plasma IL-17A levels in PD patients, but research on alterations to Th17 cells occurring in the SN of PD patients is lacking. In PD model mice, an increase in CD4^+^ T cells has been observed in the SN pars compacta, with these cells expressing RORγt, a transcription factor specific to Th17 cells. This suggests that Th17 cells migrated into and accumulated in the damaged brain of the PD model mice (Liu et al., 2016). Additionally, elevated IL-17A levels have been reported in the SN and peripheral blood of MPTP model mice (Liu et al., 2019b), and this may be associated with the breakdown of the BBB in brain regions relevant to PD (Liu et al., 2019b). When the BBB is compromised, IL-17A-producing cells can migrate from the bloodstream to the brain, further exacerbating BBB damage (Liu et al., 2019b). Moreover, Th17 cells may contribute to BBB damage, as research using an MS model has demonstrated that Th17 lymphocytes express granzyme B, which can migrate to BBB endothelial cells, induce neuronal death, and stimulate CNS inflammation by attracting CD4^+^ lymphocytes (Kebir et al., 2007).

The increase in Th17 cells may be attributed to the presence of α-syn and its induction of RORC transcription in circulating Th17 cells, which promotes their differentiation in PD (Li et al., 2023b). In addition to the effects of α-syn on Th17 cells, the interaction between microglia and Th17 cells is closely related to the onset of PD. Microglia activate intracellular inflammatory pathways, induce the release of proinflammatory cytokines, and promote the differentiation of CD4^+^ T cells into the proinflammatory Th1 and Th17 subtypes (Su and Zhou, 2021; **Figure 2**).

Th17 cells have been associated with neuronal loss in PD (Li et al., 2023b). In MPTP-induced PD mouse models, α-syn-stimulated Th17 cells reportedly contributed to neuronal death in the SN (Reynolds et al., 2010). A recent study reported that midbrain neurons generated from PD-induced pluripotent stem cells experienced increased mortality due to IL-17R overexpression and NF-κB activation following their coculture with T lymphocytes or the addition of IL-17 (Sommer et al., 2019). In addition, blocking IL-17 or IL-17R, or adding anti-IL-17 antibodies, reduced the amount of neuronal death (Sommer et al., 2019). Liu et al. (2016) demonstrated that the direct neuronal injury caused by Th17 cells is mediated by interactions involving intercellular adhesion molecule-1, the lymphocyte function-associated antigen-1 receptor found on DA neurons, and lymphocyte function-associated antigen-1 expressed on Th17 cells (Liu et al., 2016). In addition to directly harming neurons, Th17 cells can also cause indirect harm by promoting the activation of glial cells. Another study indicated that IL-17A exacerbates DA neuronal loss only in the presence of microglia, and silencing the IL-17A receptor in microglia reverses this effect (Liu et al., 2019b). The TNF-α released by microglia mediates the synergistic damage caused to DA neurons through IL-17A (Liu et al., 2019b). IL-17A acts on IL-17A receptors on microglia cells to enhance their TNF-α release, subsequently leading to DA neuronal death (Liu et al., 2019b). In a MPTP-induced mouse model of PD, the chemokine regulated on activation, normal T cell expressed and secreted (RANTES) was shown to promote T cell infiltration and result in prolonged DA neuronal death. Furthermore, PD patients have higher serum levels of RANTES and IL-17 (Dutta et al., 2019), and the RANTES-Th17 pathway is crucial to the degeneration of DA neurons in PD. The C–X–C chemokine receptor type 4 (CXCR4) and CXCL12 signaling axis are known to regulate Th17 cells in the context of neuronal degeneration in PD (Gate et al., 2021). Several CXCR4 antagonists are currently being investigated in clinical studies as ways to prevent pathological Th17 cell trafficking to the brain in PD patients (Stone et al., 2007). Taken together, these findings suggest that IL-17A plays a significant role in the neuroinflammation that leads to the neuronal death associated with PD.

Some PD symptoms have an association with the frequency of Th17 cells in the peripheral blood of patients. PD patients with symptoms of constipation have a significantly higher Th17 frequency than those without constipation (Chen et al., 2015b). Patients with early-stage PD have an increased number of proinflammatory Th17 cells, and those with higher Th17 cell counts have lower Unified PD Rating Scale (UPDRS) scores than patients with lower counts (Yan et al., 2021). Additionally, there is a correlation between the frequencies of Th1 and Th17 cells and UPDRS score components (Yang et al., 2021a).

#### Role of regulatory T cells in Parkinson’s disease

Tregs constitute 5%–10% of all CD4^+^ T cells in the immune system and play a crucial role in regulating CD4^+^ T cell immunity (Sakaguchi et al., 2008). As Th17 cells and Tregs have opposing effects, Tregs function in PD in a manner that contrasts with that of Th17 cells.

Tregs inhibit both immune cell activation and neuroinflammation (Park et al., 2023). The immunomodulated expansion of Tregs in PD mice was observed to reduce the populations of CD4^+^ and CD8^+^ T cells in the nigrostriatal tract at an early stage of disease development (Badr et al., 2022). Furthermore, the administration of granulocyte-macrophage colony-stimulating factor mRNA to PD model mice promoted the generation of Tregs, which in turn reduced microglial activation and increased the levels of anti-inflammatory mediators (Olson et al., 2020, 2021b). These findings suggest that the expansion of Tregs may slow the degeneration of DA neurons and mitigate neuroinflammation in PD animal models. Notably, the adoptive transfer of CD3-activated Tregs into PD model mice protected the nigrostriatal system by more than 90% (Reynolds et al., 2007). Tregs inhibit neuronal degeneration by reducing the Th17 immunological response (Reynolds et al., 2010). The interactions between the CD45 expressed on Tregs and galectin-1 on DA neurons mediate neuroprotection by reducing microglial activation, preventing neuronal apoptosis and promoting neural healing (Huang et al., 2019). Therefore, increasing Tregs in PD patients may represent a potential strategy for treating PD by ameliorating neuroinflammation. The percentage of Tregs in PD mice and patients remains unclear. Two reports indicate there is a decrease in Tregs among PD patients (Chen et al., 2015a; Kustrimovic et al., 2018), while a previous study has suggested that PD patients may have a higher number of Tregs in their serum than healthy controls (Rosenkranz et al., 2007). In patients with PD, the ability of Tregs to suppress T effector (Teff) cells is impaired, and this dysfunction is related to both the pathobiology and the severity of the disease (Saunders et al., 2012). As discussed earlier, α-syn-induced RORC transcription stimulates Th17 cell development while destabilizing Tregs (Li et al., 2023b), and the damage to Tregs and loss of DA neurons can be mitigated by RORC inhibitors (Li et al., 2023b). Karaaslan et al. (2021) demonstrated that CD49d expression is enhanced in patients with PD, who also have a higher frequency of CD49d^+^ Tregs in their peripheral blood (Karaaslan et al., 2021). Furthermore, PD patients with a higher frequency of CD49d^+^ Tregs tend to have lower UPDRS scores (Karaaslan et al., 2021). Although CD49d+ Tregs can inhibit the development of Teff cells, their suppressive capacity is significantly lower than that of CD49d^−^ Tregs (Kraczyk et al., 2014). Kraczyk et al. (2014)’s findings suggested that the number of Tregs with high suppressive capacity is lower in patients with PD, indicating that dysfunctional Tregs may play a role in PD pathogenesis. The loss of Treg inhibitory activity may also contribute to the widespread systemic proinflammatory response observed in PD (Thome et al., 2021). Moreover, Treg dysfunction is closely associated with the non-motor symptoms of PD. In patients with more severe cognitive impairment, there is an increase in Th1 cells and activated Tregs, accompanied by a decrease in resting Tregs. This discordance between Tregs and Th1 cells may accelerate the onset of cognitive impairment in PD patients (Magistrelli et al., 2020). Overall, Treg dysfunction may be intricately linked not only to the initiation of PD but also to its progression. Numerous abnormalities in Tregs have been documented in PD, providing data to support the importance of neuroinflammation. Further research is needed to clarify how Tregs are affected in PD.

#### Other CD4^+^ T cells in Parkinson’s disease

In addition to Th17 cells and Tregs, Th1 and Th2 cells also play crucial roles in the neuroinflammation associated with PD. The Th1 subtype predominates the CD4^+^ T cell subset in the peripheral blood of patients with PD, indicating that Th1 cells may contribute significantly to the pathophysiology of PD. Given that Th1 and Th2 cells typically have opposing effects, alterations to their numbers in PD are likely to produce a range of diverse effects on the disease process.

In both PD patients and PD animal models, there is an observed overabundance of Th1 cells compared to Tregs, resulting in an imbalance that is thought to contribute to the development of PD (Li et al., 2021; Liu et al., 2022). Specifically, in patients with PD, there is a decrease in circulating Th2 cells, Th17 cells, and Tregs, which leads to a relative increase in Th1 cells and elevated Th1/Th2 and Th1/Th17 ratios (Kustrimovic et al., 2018). Given that Th1 cells are proinflammatory, their increased presence may exacerbate neuroinflammation in PD. However, it is worth noting that a previous study reported lower overall percentages of Th cells, particularly Th1 cells, in the peripheral blood samples from patients with PD (Niwa et al., 2012).

Activated Th2 cells secrete various cytokines, including IL-4, IL-9, and IL-10. The IL-4 and IL-13 produced by Th2 cells typically exert anti-inflammatory effects by inhibiting the proliferation of Th1 cells. Additionally, IL-10 is known for its anti-inflammatory properties (Mosser and Zhang, 2008) and has been shown to prevent LPS-induced DA neuronal death in the context of PD (Arimoto et al., 2007). In a recent analysis of PD samples, Th2 cells demonstrated a significant population decrease (Dong et al., 2023). In contrast, Yan et al. (2021) reported that the peripheral blood of PD patients contained a significantly higher number of Th2 cells that produce IL-4.

Although there are conflicting reports regarding the subtypes of CD4^+^ T cells at play in PD, it is clear that the abnormalities observed in these cell populations contribute to neuroinflammation, ultimately leading to the death of DA neurons.

## Neuroinflammation Mediated by Immune Cells: Focusing on the Core Functions of T Cells and Their Interactions With Other Immune Cells in Alzheimer’s Disease

AD is a progressive neurodegenerative disorder and the most common form of dementia among older individuals. The two primary pathological features of AD are amyloid plaques and neurofibrillary tangles. Amyloid plaques are predominantly composed of amyloid-beta protein (Aβ), while neurofibrillary tangles mainly consist of hyperphosphorylated tau protein (Zheng and Wang, 2024). Several factors contribute to the eventual death of nerve cells and the loss of brain tissue in patients with AD, including neuroinflammation, changes in intestinal microbial composition, genetic and environmental influences, synaptic dysfunction, and neurotransmitter imbalances (Khan et al., 2020). Among these factors, neuroinflammation is considered a key element in the pathophysiology of AD (Irwin and Vitiello, 2019).

In AD, the innate immune system is more likely to be activated, while the adaptive immune system is suppressed, leading to pathological damage and cognitive impairment. Innate immune cells, such as monocytes, M2 macrophages, and neutrophils, show greater degrees of infiltration in the brains of patients with AD than adaptive immune cells such as plasma cells, CD8^+^ T cells, Tfh cells, and activated NK cells (Jin et al., 2022). In AD transgenic mouse models that lack adaptive immune cells, such as T cells, B cells, and NK cells, higher levels of Aβ deposition and increased neuroinflammation have been observed (Marsh et al., 2016). Epidemiological evidence and preclinical research further support the idea that AD is primarily driven by an overactive innate immune system (Heneka et al., 2015). Therefore, restoring the repressed innate immune response while appropriately inhibiting the overactivated adaptive immune response could represent the most promising therapeutic strategy for AD. In this context, we next review the roles of innate immune cells, such as microglia, astrocytes, and NK cells, as well as adaptive immune cells, particularly B cells and T cells, in AD-related neuroinflammation.

### Crucial contribution of microglia and their interaction with T cells to the neuroinflammation associated with Alzheimer’s disease

The development of AD is significantly influenced by microglia, which serve a dual function. As individuals age, the function and characteristics of microglia in the brain dynamically change. In AD, Aβ protein accumulates and is typically cleared by microglia. During the early stages of AD, protective microglia are activated to eliminate Aβ; however, this clearance process eventually fails. As the disease progresses, the activation of harmful pro-inflammatory microglia gradually increases and is positively correlated with the Aβ load (Fan et al., 2017).

Recent research has identified a specific type of microglia, known as “dark microglia” (DM), which exhibit a unique dark appearance and distinct ultrastructural properties (Bisht et al., 2016). DM are primarily found in the peripheral regions of dystrophic neurites and Aβ plaques in postmortem brain tissues from patients with AD (St-Pierre et al., 2022). Microglia associated with amyloid plaques display notable transcriptomic alterations compared to those without amyloid plaques (Grubman et al., 2021). This suggests that microglia in different states vary in their regulation of gene expression and other functional aspects, impacting their role in AD.

In addition to Aβ, the accumulation of tau protein also promotes microglial overactivation and inflammation. Tau protein selectively activates TLR2, leading to microglial inflammation (Dutta et al., 2023). Tau accumulation also increases the activation of NF-κB in microglia, which results in neuronal damage and cognitive impairments (Wang et al., 2022a). Prolonged microglial activation leads to the continuous release of inflammatory factors, diminishing their ability to engulf and degrade neurotoxins. This decline in capacity ultimately drives the progression of AD, resulting in further Aβ accumulation, accelerated tau propagation, and increased neuronal death.

Microglia can also influence the progression of AD by modulating T lymphocytes; for example, they have the capacity to induce apoptosis in Teff cells within tissues, leading to T cell tolerance or unresponsiveness (Ford et al., 1996). Conversely, microglia can activate T cells and enhance immune responses by phagocytosing Aβ and presenting it to T cells. Single-cell RNA sequencing analyses suggest that the number of chemokine ligand-receptor interactions between microglia and CD8+ T cells is greater than that observed with other cell types, indicating the potential role of cell-to-cell communication and collaboration during both physiological and pathological processes (Su et al., 2023). Moreover, a separate study has shown that CD8^+^ T cells significantly interact with Aβ-reactive microglia (Yamakawa and Rexach, 2024). When microglia are depleted, T cell infiltration into the brain is virtually eliminated, and conversely, the activation of microglia is hindered when T cells are depleted (Chen et al., 2023b). This demonstrates how innate and adaptive immune cells can communicate with one another.

### Crucial contribution of astrocytes and their interaction with T cells to the neuroinflammation associated with Alzheimer’s disease

In the early stages of AD, there is a proliferation of astrocytes prior to the formation of Aβ plaques (Rodriguez-Vieitez et al., 2015a, b). Research has shown that individuals with familial AD mutations exhibit signs of astrocyte hyperplasia even while asymptomatic (Rodriguez-Vieitez et al., 2016). Under pathological conditions, reactive astrocytes undergo significant morphological and functional changes, which manifest as the increased release of neurotoxic factors and cellular enlargement. These reactive astrocytes can clear Aβ through various mechanisms, and enhancing their phagocytic activity may offer a potential therapeutic avenue for AD (Wyss-Coray et al., 2003; Gomez-Arboledas et al., 2018). Recent findings indicate that the relationship between Aβ and early tau phosphorylation in preclinical AD is influenced by the increased presence of reactive astrocytes (Bellaver et al., 2023). Specifically, the release of glial fibrillary acidic protein (GFAP) and YKL-40 by astrocytes plays a critical role in the disease’s progression. Elevated plasma GFAP levels may maintain an astrocyte-mediated neuroprotective equilibrium during preclinical stages, counteracting Aβ aggregation through glial homeostatic mechanisms. In contrast, CSF YKL-40 elevation emerges later in the neurodegenerative cascade, specifically associated with tau-mediated axonal injury and hyperphosphorylation (Pelkmans et al., 2024). When neurons produce excess Aβ peptides, the NF-κB pathway can become activated in astrocytes, leading to an increase in complement C3 expression, which negatively affects both neurons and microglia. This cascade can result in microglial activation and neuronal damage, both of which are critical events in the development of AD (Lian et al., 2016). Additionally, astrocytes can detect Aβ aggregates through TLR4 and the receptor for advanced glycation end products, subsequently activating downstream target genes and producing various associated molecules (Paudel et al., 2020). Consequently, neuroinflammation is exacerbated, contributing to the progression of AD.

Although less well documented, there is some evidence that interactions between astrocytes and T cells are disease mediators. The numerous cytokines and chemokines released by astrocytes impact T cell activity. Additionally, the active migration of white blood cells to the CNS is influenced by the interactions between astrocytes and activated peripheral immune cells. Glial cells are effective APCs for Aβ-specific Th1 and Th17 cells. Co-culturing these Aβ-specific Th1 or Th17 cells with glial cells can increase relevant inflammatory responses and molecular expression, whereas Th2 cells can suppress some inflammatory responses and associated molecular expression (Steele and Robinson, 2012). Moreover, the number of CD4^+^ T cells increases when they are co-cultured with Aβ-pretreated astrocytes (Spampinato et al., 2020).

In general, astrocytes transition from their initial state to a reactive state following prolonged exposure to Aβ. They shift from being cells that assist in neuronal metabolism to those exhibiting inflammatory characteristics. This alteration is likely to impair the initially beneficial interactions between astrocytes and neurons, preventing astrocytes from supporting neurons as they normally should.

### Dual regulatory role of natural killer cells in the neuroinflammation of Alzheimer’s disease

Although NK cells tend to proliferate with aging, their effectiveness declines. This discrepancy between quantity and function means that, while NK cells may become more numerous overall, their ability to eradicate tumor and infected cells is diminished (Brauning et al., 2022). Because age is the primary risk factor for AD, the subpopulation of NK cells is particularly important in this context.

The role of NK cells in AD remains a topic of debate. Some studies have reported reduced NK cell cytotoxicity, while others implicate cytotoxic NK activity in the pathogenesis of AD. Single-cell analyses have revealed elevated proportions of NK cells in AD microenvironments (Li et al., 2024b). Additionally, analyses of the CSF from patients with AD have shown that NK cell expansion correlates with tau pathology (Busse et al., 2021), and this may be linked to the spontaneous release of pro-inflammatory cytokines, such as TNF-α and IFN-γ, by NK cells (Solerte et al., 2000). Furthermore, investigations of transgenic AD mouse models have demonstrated the enhanced pro-inflammatory properties of NK cells (Zhang et al., 2020). In these models, a depletion of NK cells significantly reduced neuroinflammation but did not affect Aβ accumulation (Zhang et al., 2020). Conversely, Qi et al. (2022) found that both the quantity and percentage of NK cells were decreased in the blood of patients with AD, along with reductions in cytotoxic and adaptive NK cell subpopulations. Although there is no conclusive evidence, this decline may be due to the activation of NK cells and their migration to different organs in AD patients with AD (Lu et al., 2021; Qi et al., 2022). Similarly, research has shown that plasma from AD patients contains a significantly smaller percentage of NK cells than that of individuals without dementia (Zeng et al., 2024). Moreover, a negative correlation was reported between the number of activated NK cells and the severity of neurofibrillary tangles caused by tau protein misfolding in patients with AD (Qian et al., 2022). Other studies, however, have reported no significant alterations in NK cell populations in the plasma of patients with AD (Richartz-Salzburger et al., 2007; Huang et al., 2022b).

In summary, the involvement of NK cells in the neuroinflammation associated with AD is complex. While they may play a role in regulating neuroinflammation through the pathways mentioned, their specific activity is still debated.

### Crucial contribution of B cells and their interaction with T cells to the neuroinflammation associated with Alzheimer’s disease

There has been growing interest in the roles of B lymphocytes and plasma cells in AD, and research has revealed that B cells play a dual role. Although they are believed to express cytokines that ameliorate AD and create antibodies against Aβ (Baulch et al., 2020), they are also believed to worsen AD symptoms.

According to several research efforts, patients with AD exhibit significantly lower blood B lymphocyte counts (Richartz-Salzburger et al., 2007; Xiong et al., 2021; Song et al., 2022). Conversely, results have been obtained showing a noticeably higher proportion of B cells in the plasma of patients with AD than healthy controls (Huang et al., 2022b). In AD mice, IgG levels in the cerebral cortex and hippocampus were elevated, and there was an increased number of B lymphocytes in their brains (Kim et al., 2021). This increase in B cells may offer some benefit in preventing AD pathogenesis. Feng et al. (2023) reported B cell accumulation was seen in the meninges and frontal cortex of AD mice. These B cells release more IL-35, and when injected into AD animals, reduced Aβ load and ameliorated cognitive dysfunction. Furthermore, a lower risk of AD has been linked to CD25 expression on the surface of switched memory B cells (Zhang et al., 2024b). B cell depletion has been shown to exacerbate cognitive decline and increase Aβ burden in early AD models (Xiong et al., 2021). However, under certain circumstances, an increase in B cells can negatively impact the onset of AD. A positive correlation has been observed between the amount of brain Aβ deposition and the rise in B lymphocytes (Park et al., 2022). In AD mouse models, a therapeutic reduction in B lymphocytes at the onset of the illness was found to lower the Aβ plaque load and decrease the activation of disease-associated microglia (Kim et al., 2021).

The interaction between T cells and B lymphocytes is a significant factor in neuroinflammation during the progression of AD. Notably, IL-21, a hallmark cytokine produced by Tfh cells, is markedly elevated in AD patients. This increase in IL-21 has been positively correlated with elevated IgG levels (Baulch et al., 2020), suggesting that T cells are influential in both antibody synthesis and the activation of B cells. This interplay highlights the importance of T cell–B cell interactions in the neuroinflammatory processes associated with AD.

Overall, while B cells may play a beneficial role in AD under certain circumstances, their increased quantity is also likely to contribute to the progression and worsening of the disease through various pathways. This indicates that the potential adverse effects of B cells must be carefully considered to ensure a more balanced and effective approach when developing treatment regimens for AD.

### Multifaceted roles of T cells in neuroinflammation and the pathophysiology of Alzheimer’s disease

During the progression of AD, T cells can move from the peripheral circulation to the CNS to participate in the neuroinflammatory response. T cells are implicated in the neuroinflammatory response in AD, according to numerous clinical investigations and animal tests. Research on T cells in AD is contentious. T-cell changes may be influenced by a variety of factors, so T-cell subset analysis is necessary.

#### CD8^+^ T cells in Alzheimer’s disease

A 2022 meta-analysis confirmed elevated peripheral CD8^+^ T cells in AD patients (Huang et al., 2022b). In the hippocampal parenchyma of AD patients, more CD8^+^ T lymphocytes are present (Unger et al., 2020). A single-cell sequencing investigation revealed that CD8^+^ T cells with the same clonotype in both blood and CSF may play a significant role once they enter the brain (Gate et al., 2020). CD8^+^ T-cell infiltration into AD cultures resulted in microglial activation and neuroinflammation in a three-dimensional human neuroimmune axis model (Jorfi et al., 2023). CD8^+^ T-cell infiltration is induced by the CXCL10-CXCR3 pathway (Jorfi et al., 2023). Compared with people without dementia, those with AD-induced dementia have many more CD8^+^ T cells in their brains (Yamakawa and Rexach, 2024). This finding implies that CD8^+^ T lymphocytes worsen neuroinflammation and accelerate AD development. However, investigations have revealed that CD8^+^ T cells expressing distinct markers have diverse impacts on AD. According to research in AD mouse models, CD8^+^ T cells in the brain are genetically characterized as tissue-resident memory (Trm) T cells (Altendorfer et al., 2022). Neurons gradually die as a result of CNS inflammation caused by persistent CD8^+^ Trm cells (Kimura et al., 2024). Furthermore, the brains of AD animal models also exhibit a notable increase in PD-1^+^ CD8^+^ T lymphocytes, which can decrease Aβ plaque deposition by lowering the inflammatory state of microglia (Su et al., 2023). In the brain, CXCR6 is essential for CD8^+^ T-cell accumulation, retention, and clonal growth. Following CXCR6 loss, brain PD-1^+^ CD8^+^ T-cell activity is compromised, microglia produce more proinflammatory cytokines, and neuroinflammation is exacerbated (Su et al., 2023). Furthermore, because CD8^+^ T cells form tight complexes with microglia near Aβ plaques in both human and AD animal brains, CD8^+^ T cells may intensify mouse AD-like symptoms by targeting disease-associated microglia (DAMs) (Wang et al., 2024b). Wang et al. (2024c) reported that three types of T-cell subsets with effector functions are primarily active in the peripheral blood and CSF of AD patients: CD8^+^ TEMRA (terminal differentiation effector memory T cells), CD8^+^ TEM (effector memory T cells), and CD4^+^ TEMRA cells. Since CD8^+^ TEMRA cells in AD patients’ CSF build up prior to the onset of dementia, they may be involved in the early stages of AD through the expression of markers of T-cell aging (van Olst et al., 2024). There is a decrease in communication between CD8^+^ TEMRA cells and other T cells (Wang et al., 2024c). This breakdown of intercellular communication may make it more difficult for other T cells to regulate CD8^+^ TEMRA cells, which could ultimately cause CD8^+^ TEMRA cells to proliferate and malfunction abnormally, worsening AD. GFAP and neurofilament light chain (Nf-L) expression are favorably connected with the number of CD8^+^ TEMRA cells in the peripheral blood of AD patients (Winford et al., 2024). GFAP serves as an indicator of astrocyte activity. Nf-L is expressed mainly in axons and is released into the blood circulation system and CSF when an axon is damaged, making it a useful signal for determining whether an axon is damaged. Therefore, the accumulation of CD8^+^ TEMRA cells is likely associated with neuronal injury and neuroinflammation.

#### CD4^+^ T cells in Alzheimer’s disease

Mendelian randomization links elevated CD4^+^ T-cell counts to increased AD risk (Fani et al., 2021). CD4 memory-related T-cell infiltration is increased in AD patients (Yang et al., 2024). There is a notable increase in a subset of CD4^+^ T cells with high IFN responsiveness in the blood of patients with sporadic early-onset AD (Sirkis et al., 2024). Cognitive deterioration in AD patients was linked to a large increase in CD4 Tc cells in peripheral blood mononuclear cells (Chen et al., 2024). A subset of CD3^+^ T cells are CD4^+^ CD8^dim^ T cells, which are produced from double-positive T cells (Suni et al., 2001). Zhang et al. (2024a) revealed that CD4^+^ CD8^dim^ T lymphocytes are significantly associated with the progression of AD. In AD, there is considerable communication between CD4^+^ and CD8^+^ T cells. CD4^+^ T cells facilitate the recruitment and adherence of CD8^+^ T cells, allowing T cells to enter the brain parenchyma (Weng et al., 2024). However, Huang et al. (2022b) revealed no difference in peripheral blood CD4^+^ T cells between healthy controls and AD patients.

In AD patients, an increase in Th1 cytokines fosters an inflammatory milieu (Vasantharekha et al., 2024). Compared with healthy controls, AD patients have an increased Th1/Th2 ratio (Lewis et al., 2023). Th1 cells, which encode T-bet transcription factors, are crucial for the type 1 immune response and are regulated by TBX21. T-bet encourages Th1 cells to release IFN-γ, which causes inflammation, and directs T cells to approach the site of inflammation. The TBX21 immune gene mRNA level is markedly elevated in the peripheral blood of patients with late-onset AD (Fatemi Langroudi et al., 2023). Th17 cells play a key role in the development of AD. Early in AD, IL-17 starts to play a part, and when cognitive decline starts in female mice, IL-17-producing T cells start to accumulate in their brains and meninges (Brigas et al., 2021). Adoptive transfer of Aβ-reactive Th1 and Th17 cells accelerates memory loss, systemic inflammation, and microglial activation in AD model mice (Machhi et al., 2021). CD4^+^ T cells that suppress IL-17a expression in the peripheral circulation preserve or even increase microglial activity and support Aβ clearance and effects in the brain (Hao et al., 2024).

Tregs play a major protective role in the progression of AD (Zuo et al., 2024). Tregs attenuate pathology in AD models by suppressing proinflammatory factors (IL-1α/β, IL-6), complement pathways (C1q, C4), TLR signaling (TLR3/4/7), and microglial markers (CD14/Trem2) (Faridar et al., 2022). Furthermore, Tregs control the responsiveness of AD astrocytes by promoting A2-like subpopulations and suppressing A1-type astrocytes (Stym-Popper et al., 2023). Treg and Th17 cell equilibrium in the brains and peripheral tissues of AD mice quickly becomes imbalanced as the disease progresses (Yuan et al., 2023). The total number of peripheral Tregs and the number of resting Tregs are considerably lower in individuals with middle-stage AD than in healthy people (Ciccocioppo et al., 2019). A comparison of various groups revealed that patients with mild cognitive impairment had the highest percentage of Tregs in their peripheral blood, followed by healthy control subjects, whereas patients with moderate to severe AD-related dementia had the lowest percentage of Tregs (Fu et al., 2020). This finding indicates that the immunological reserve of those suffering from AD-related dementia is compromised. Tregs are promising for treating AD because of their protective effects. Low doses of recombinant human IL-2 administered peripherally can reduce neuroinflammation, correct the middle-stage imbalance between the Treg and Th17 subgroups, and specifically control the number of Tregs (Yuan et al., 2023). Treg immunotherapy that targets specific antigens has demonstrably shown promise in treating AD (Yang et al., 2022; Yeapuri et al., 2023).

**Figure 3:**
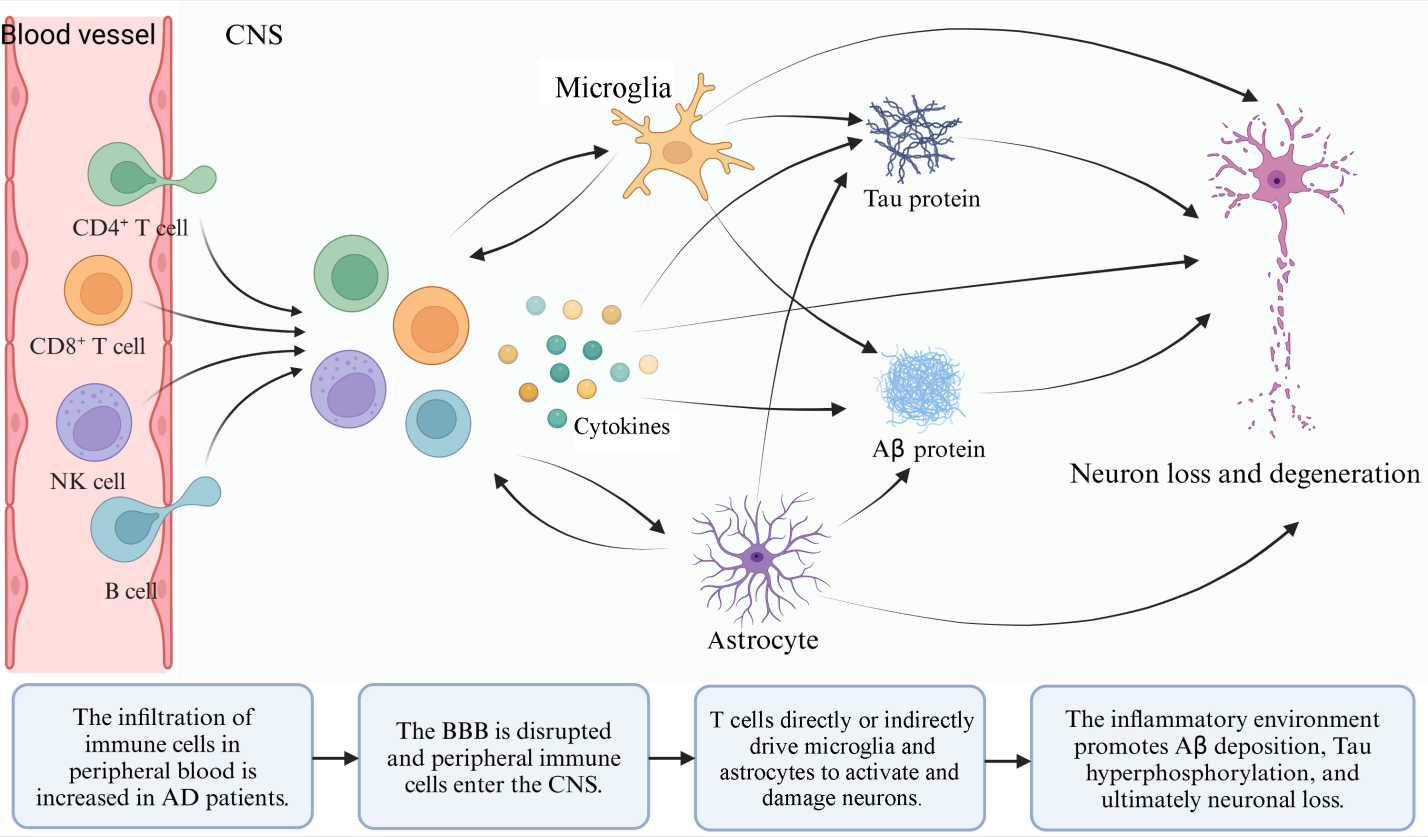
T-cell regulation of neuroinflammation in AD. The BBB is disrupted in the pathological state of AD when immune cells invade the periphery and produce a number of cytokines. Immune cells that enter the CNS emit a range of cytokines. A number of intricate pathogenic cascades are set off by the interaction between these immune cells and the cytokines they release and microglia and astrocytes in the CNS. The Aβ protein progressively accumulates, and the tau protein undergoes aberrant phosphorylation throughout this process, ultimately resulting in the death and functional deterioration of nerve cells. Aβ: Amyloid-β peptide; AD: Alzheimer’s disease; BBB: blood‒brain barrier; CNS: central nervous system; NK: natural killer cell.

The role of T cells in AD is intricate and vital, and each type of T-cell has distinct symptoms and impacts. They either accelerate inflammation, harm neurons, or have immunomodulatory and neuroprotective functions (**Figure 3**). They are also strongly linked to the clinical features of AD, such as Aβ accumulation and pathological alterations in the tau protein. Immunoregulatory therapy and cell therapy, which modulate the balance of T-cell subsets and alleviate the inflammatory milieu in the brain, can help slow the progression of AD.

## Role and Relationship of Neuroinflammation With the Involvement of Immune Cells in Other Neurodegenerative Diseases: A Comprehensive Analysis of the Dominant Role of T Cells and the Interactions Among Immune Cells

### Regulation of neuroinflammation by immune cells: Core role of T cells and their synergistic mechanisms in amyotrophic lateral sclerosis

ALS is a degenerative disease of the nervous system that affects motor neurons. There is no known cure for ALS, and the main goal of treatment is to maximize the patient’s quality of life through palliative and supportive measures. ALS has a complex pathophysiology that includes DNA and RNA damage, mitochondrial malfunction, neuroinflammatory responses, and hereditary variables, and the immune system is a key player in this process. Motor neurons in ALS patients and mice degenerate when the blood‒spinal cord and BBB are damaged, and the development of NDD and the progression of the disease are delayed when barrier integrity is restored (Garbuzova-Davis et al., 2012; Winkler et al., 2014). The neuroinflammation associated with ALS involves peripheral immune cell infiltration and glial cell activation. The quantity of invading white blood cells is successfully decreased by limiting the transfer of peripheral immune cells to the CNS (Garofalo et al., 2022).

#### Microglia and neuroinflammation in amyotrophic lateral sclerosis

In the context of neuroinflammation in individuals with ALS, microglia are crucial (Shen et al., 2024). A transcriptomic study revealed a substantial increase in microglia in the motor cortex of ALS patients, and certain subpopulations presented gene expression traits resembling those of DAM (Dols-Icardo et al., 2020). Postmortem analyses and PET imaging confirmed that microglial activation is correlated with ALS progression (Brettschneider et al., 2012; Tondo et al., 2020). Similar to PD and AD, ALS may cause microglia to change phenotypically. Microglia isolated from early-stage ALS mice exhibit an M2 phenotype that protects motor neurons, whereas microglia derived from end-stage ALS mice present a neurotoxic M1 phenotype (Liao et al., 2012). When neuroprotective microglia are transplanted into ALS model mice, microglia and astrocytes proliferate, and neuronal cell death is suppressed, greatly enhancing the motor function and survival rate of the spinal cord in ALS model mice (Kobashi et al., 2022). In addition to the conventional M1 and M2 phenotypes, microglia can also be classified into two subgroups, TMEM119^+^ and TMEM119-microglia, according to their pathophysiology. TMEM119^+^ microglia express the microglial activation marker CD68 and exhibit endothelial activation. Research has shown that MEM119^+^ microglia are prevalent in the motor cortex’s subcortical white matter and the motor area of ALS patients, suggesting that inflammation is present in ALS lesions (Togawa et al., 2024). Nevertheless, DAM suppressed TMEM119 expression, which suggests that TMEM119^+^ microglia induce inflammatory neurodegeneration that is independent of DAM (Takahashi, 2023). In addition to variations in quantity and phenotype, microglial function varies during the ALS disease state. In the ALS animal model, chronically activated microglia gradually develop unique protein profiles and further decline in immunological function, eventually becoming a cell type with low immune efficacy (Barreto-Núñez et al., 2024). The mitochondria of microglia are crucial; when they breakdown or become damaged, negative astrocyte reactions may occur, leading to neuroinflammation (Joshi et al., 2019). Furthermore, neuronal degradation is also linked to microglia-mediated pyroptosis activation in ALS (Van Schoor et al., 2022).

According to the aforementioned research, the role of microglia in ALS pathology is crucial to this process, and their reactive activation might be a major contributing component to disease progression. Microglial activation signatures serve as biomarkers for monitoring antineuroinflammatory therapies in individuals with ALS (Quek et al., 2022).

#### Astrocytes and neuroinflammation in amyotrophic lateral sclerosis

In addition to microglia, astrocytes are also believed to play a significant role in neuroinflammation in ALS. An RNA sequencing analysis of autopsy samples revealed that ALS patient samples presented substantially greater astrocyte gene expression than healthy controls did (Humphrey et al., 2023). Astrocytes transition from neuroprotective to neurotoxic states during ALS progression (Ziff et al., 2022). In individuals with ALS, astrocytes derived from induced pluripotent stem cells (iPSCs) are dysfunctional and display a phenotype associated with an immunological response (Velasquez et al., 2024). One important aspect of ALS neuroinflammation is the growth of reactive astrocytes (Stoklund Dittlau and Van Den Bosch, 2023). One of the most significant biomarkers of reactive astrocytes in neuroinflammation is the action site of monoamine oxidase B (MAO-B), which is found on the outer membrane of astrocyte mitochondria in the human brain (Saura et al., 1996). ALS patients have more astrocytes in their spinal cords, and these reactive astrocytes have higher levels of MAO-B (Ekblom et al., 1994). Inhibiting astrocyte reactivity attenuates disease progression in ALS models (Guttenplan et al., 2020). Therefore, addressing astrocyte reactivity and dysfunction may help stop motor neuron loss and lessen the pathogenic process of ALS. In addition to their number, astrocyte functions can be altered. By controlling glutamate and ion homeostasis, removing oxidative stress, supplying energy support, and participating in synaptic development, astrocytes contribute significantly to the maintenance of a healthy CNS environment. Decreased introns in reactive astrocytes in ALS are linked to increased transcription during cell adhesion, the stress response, and immunological activation (Ziff et al., 2021). In ALS, extracellular vesicles secreted by astrocytes either mediate or worsen neuroinflammatory reactions (Upadhya et al., 2020). Astrocytes typically use excitatory amino acid transporter 1 (EAAT1) and EAAT2 to remove glutamate from the synaptic gap for homeostasis. However, in ALS, astrocytic glutamate transporter activity is impaired, causing excess extracellular glutamate, which triggers excitotoxicity and neuronal death (Provenzano et al., 2023). In ALS, astrocyte mitochondrial failure can also trigger neuroimmune responses, which can harm neurons (Zhao et al., 2022).

In summary, astrocytes are essential to the pathophysiology of ALS. Further investigations into the function of astrocytes in ALS could lead to novel therapeutic approaches.

#### Natural killer cells and neuroinflammation in amyotrophic lateral sclerosis

On the basis of the variation in CD16 and CD56 expression, NK cells in peripheral blood are separated into two primary subgroups: CD16^+^ CD56^dim^ NK cells and CD16^–^CD56^bright^ NK cells (Farag and Caligiuri, 2006). Approximately 90% of peripheral blood NK cells are CD16^+^CD56^dim^ NK cells, which are primarily in charge of cytotoxic effects through the release of granzymes and perforin. Approximately 10% of NK cells in peripheral blood are CD16^+^CD56^bright^ NK cells. Despite being rare, CD16^+^CD56^bright^ NK cells are vital for immunoregulation, as they secrete cytokines such as TNF-α, G-CSF and IFN-γ to help control the immune response (Freud et al., 2017).

NK cells have been poorly studied in individuals with ALS. In a way that is specific to age and sex, NK cells accelerate the course of ALS (Murdock et al., 2021). ALS patients have increased peripheral blood NK cell counts (Murdock et al., 2017), along with increased levels of cytotoxicity and IFN-γ release (Kaur et al., 2022). NK cells proliferate in the CNS of ALS animals and colocalize with motor neurons, causing an inflammatory response in microglia through the production of IFN-γ (Garofalo et al., 2020). Peripheral NK cell elevation in ALS patients is primarily due to CD56^bright^ subset expansion, which contrasts with CD56^dim^ reduction (Jin et al., 2020). Compared with ALS patients whose disease progresses more slowly, those whose disease progresses more quickly have fewer CD56^bright^ NK cells in their CSF (Rolfes et al., 2021). Furthermore, a decreased incidence of ALS was predicted by high expression levels of CD16^–^CD56^bright^ (Gong et al., 2022). In conclusion, a number of studies have demonstrated that CD56^bright^ NK cells can migrate inside the CNS of ALS patients and can be crucial for neuroprotection. By controlling the T-cell response, CD56^bright^ NK cells may provide protection for ALS patients. This regulatory capacity becomes impaired during rapid disease progression (Rolfes et al., 2021).

#### B cells and neuroinflammation in amyotrophic lateral sclerosis

The function of B lymphocytes in ALS is not well understood. Research has failed to identify intracerebral or perivascular B-cell infiltration in ALS patients (Engelhardt et al., 1993). In recent years, Kononets et al. (2022) studied blood markers of ALS patients and reported that B lymphocyte counts were lower in almost all patients (94.5%). ALS-like illnesses develop in transgenic mice devoid of B cells (Naor et al., 2009). Furthermore, ALS patients may have defective B lymphocytes due to notable alterations in IgG subclass expression (Ostermeyer-Shoaib and Patten, 1993). Intravenous B cells have neuroprotective properties and improve the prognosis of ALS patients (Sîrbulescu et al., 2024). Although recent pertinent research has indicated that B cells’ primary function may be to slow the progression of ALS, it is important to remember that B cells may also worsen the disease’s pathological alterations by encouraging inflammation.

#### T lymphocytes are key factors in the pathophysiology of amyotrophic lateral sclerosis

When white blood cell composition was investigated in individuals with ALS, the T-cell subsets in the blood and CSF significantly differed. The results of T-cell subset analysis in CSF may more precisely reflect the true neuroimmune response in ALS since CSF is situated closer to injured motor neurons (Yazdani et al., 2024). The spinal parenchyma of ALS mice is infiltrated by CD3^+^ T cells, including CD4^+^ and CD8^+^ T cells (Garofalo et al., 2022).

ALS CD8^+^ T cells are present in patients’ CSF and peripheral blood at elevated frequencies (Rolfes et al., 2021). The brain, spinal cord, and peripheral blood of ALS mice contain cloned CD8^+^ T cells (Campisi et al., 2022). ALS patients have more effector/memory CD8^+^ T cells in their peripheral blood, and these cells secrete more granzyme B and IFN-γ (Kaur et al., 2022). Furthermore, there is an increase in the number of CD8^+^ T cells that express CD95, which is linked to increased neurological impairment (Yildiz et al., 2023). A unique subset of CD8^+^ T cells known as GZMKhi CD8^+^ T cells has high levels of granzyme expression. According to Kim et al., an increase in CD8^+^GZMKhi TEMs was the most notable alteration in T-cell subsets in the CSF of ALS patients, which aided in the progression of the illness (Kim et al., 2024).

CD4^+^ T cells are frequently regarded as protective factors in ALS because they can identify antigens that APCs convey through MHC II molecules. According to Beers et al., CD4^+^ T lymphocytes may help maintain the equilibrium between glial cell cytotoxicity and nourishment, thereby offering supporting neuroprotection to the body (Beers et al., 2017). The changes in the number of CD4^+^ T cells in the peripheral blood and CSF of ALS patients are controversial. The peripheral blood CD4^+^ T cells of ALS patients either rose or fell (Mantovani et al., 2009; Murdock et al., 2017). ALS patients have increased numbers of CD4^+^ T cells in their CSF (Rolfes et al., 2021). According to a different study, ALS patients had a substantially lower percentage of CD4^+^ T cells in their CSF than controls did, and this decrease was linked to a greater degree of clinical severity in ALS patients (Kim et al., 2024). Additionally, there was a significant decrease in CD4^+^ T cells in the peripheral blood of ALS patients with cognitive impairment (Yang et al., 2021b). As the illness progresses, the phenotype of CD4^+^ T cells shifts. The phenotype of CD4^+^ T cells in advanced ALS mice is considerably altered compared with that in early ALS mice, resulting in a proinflammatory phenotype that is favorably linked with infiltrating CD8^+^ T cells (Zaccai et al., 2024). ALS patients have increased blood levels of CD57-expressing CD4^+^ T cells, a marker of severe immunological senescence of T cells that is frequently linked to increased reactivity to DNA damage and other events (Yildiz et al., 2023). With aging, tissue damage may result from cytotoxic reactions, aberrant cytokine release, and the collapse of immunologically tolerant lines (Carrasco et al., 2022). According to a Mendelian randomization study, the risk of ALS is strongly correlated with CCR7 expression on naive CD4^+^ and naive CD8+ T cells (Lu et al., 2024).

The use of CD4^+^ T cells in illness research involves considering the spectrum of various CD4^+^ T-cell subgroups. Rather than being directly tied to the general change in the CD4^+^ T-cell population, the change in the total number of CD4^+^ T cells may be related to the regulation of a particular subgroup of CD4^+^ T cells. IL-18 is a proinflammatory cytokine linked to Th1 cell development. The blood IL-18 levels are noticeably higher in ALS patients than in healthy individuals (Italiani et al., 2014). A decreased risk of death was linked to Th2 variations in the percentage of CD4^+^ T cells. The proinflammatory phenotype, which is linked to the severity and progression of the disease, replaced the initial anti-inflammatory phenotype, which is driven by Th2 cells, in the peripheral immunophenotype of ALS patients (Jin et al., 2020).

Targeting IL-17A enhances motor function and reduces neuroinflammation in mice (Limone et al., 2024). However, Tregs have demonstrated considerable therapeutic potential in ALS research. Patients with ALS who have high levels of effector T cells in their blood and CSF are less likely to survive, whereas those who have high levels of activated Treg cells are more likely to do so (Yazdani et al., 2022). There is a negative correlation between the pace of disease development and the total Treg level in the peripheral blood of individuals with ALS (Sheean et al., 2018). Passive transfer of Tregs can reduce neuroinflammation and increase longevity in a mouse model of ALS (Rajabinejad et al., 2020). Treg amplification increases the expression of neurotrophic factor genes in the spinal cord and peripheral nerves and markedly reduces the immunological reactivity of microglia and astrocytes in the spinal cord of ALS animals (Sheean et al., 2018). However, Beers et al. reported that ALS patients had impaired Treg function (Beers et al., 2017). The activation mechanism of proinflammatory microglia is impacted by malfunctioning Tregs, which increase the excitatory toxicity of ALS motor neurons (Giovannelli et al., 2020).

All possible CD4^+^ and CD8^+^ T-cell infiltration was observed in the spinal cord and the ALS disease model in mice. However, the significance of CD4^+^ and CD8^+^ T cells in the neuroinflammatory process has not been thoroughly investigated because of changes in their function as they mature.

### Regulation of neuroinflammation by immune cells: Core role of T cells and their synergistic mechanisms in multiple sclerosis

MS is a chronic inflammatory demyelinating disease of the CNS. It is characterized by an abnormal immune system that mistakenly attacks the myelin sheath in the brain and spinal cord, destroying the myelin sheath and ultimately damaging nerve signal transmission. According to estimates, 2.8 million MS cases were estimated to be prevalent globally in 2020 (Walton et al., 2020). The pathophysiology of MS, a complicated illness of the CNS, is still mostly unknown. However, the main source of neuronal injury is believed to involve immunological mechanisms, particularly those linked to aberrant lymphocyte activation.

#### Microglia and neuroinflammation in multiple sclerosis

The postmortem mononuclear RNA transcriptome of MS patients revealed significant microglial activation at the edges of MS lesions (Schirmer et al., 2019). Microglia with proinflammatory phenotypes participate in phagocytosis, oxidative damage, antigen presentation, and T-cell costimulation during the early phases of demyelination and neurodegeneration in active MS lesions (Zrzavy et al., 2017). These findings suggest that demyelination and neuroinflammation in MS are linked to microglial activation. In an EAE mouse model, microglia exhibit a transcriptional signature of cellular senescence, and these microglia have proinflammatory features that are detrimental to MS (Drake et al., 2024). Crucially, triggering receptor expressed on myeloid cells (TREMs) mediates microglial overactivation or inactivation, which exacerbates neuroinflammation and neurodegeneration in MS by releasing proinflammatory cytokines and neurotoxic substances (Farzan et al., 2025). Targeting TREM receptors specifically encourages tissue healing and suppresses detrimental proinflammatory reactions. Mitochondrial complex I sustains microglial activation in MS models, and its inhibition reduces neurotoxic damage (Peruzzotti-Jametti et al., 2024). The microglial transient receptor potential vanilloid 1 (TRPV1) pathway regulates Ca^2+^ endogenous flow, leading to neuroinflammation via activation of the NLRP3 inflammasome (Zhang et al., 2021). Receptor-interacting protein kinase 1 (RIPK1) activation in astrocytes and microglia initiates detrimental neuroinflammatory processes that aid in the progression of MS (Zelic et al., 2021). It has been demonstrated that the calc-binding protein S100A4, which acts through the Toll-like receptor 4/NF-κB pathway, promotes the microglial inflammatory response and microglia-induced neuronal death in animals and cell experiments (Jingjing et al., 2024).

In the EAE model, microglia may be able to activate the first wave of brain-producing T cells that arrive in the CNS, and this activation leads to the subsequent recruitment of T cells and dendritic cells, which then aids in broad T-cell activation, leading to demyelinating issues and degenerative disorders in the CNS (Sosa et al., 2013). Additionally, myelin-specific T cells trigger reactive microglia-like cells in lesions, which in turn release the proinflammatory cytokine IL-1β (Grebing et al., 2016). Furthermore, TNF-α produced by microglia stimulates brain endothelial cells to express transient receptor potential vanilloid-type 4 ion channel (TRPV4), which results in BBB disruption. T-cell invasion in the brain is further exacerbated by BBB disruption (Hansen et al., 2024). Cognitive difficulties in MS patients are linked to microglial phagocytosis of C1q-labeled inhibitory synapses, which can be induced by direct or indirect activation of CD8^+^ T cells (Barros et al., 2024).

#### Astrocytes and neuroinflammation in multiple sclerosis

Astrocyte phagocytosis contributes to demyelinating illness in MS. In addition to swelling and deforming when astrocytes take up myelin debris, they can also attract inflammatory cells to contribute to nerve injury (Ponath et al., 2017). Patients with relapsing-remitting multiple sclerosis (RRMS) have higher levels of GFAP in their CSF, which is strongly linked to a greater chance of RRMS progression (Azzolini et al., 2022). There is an increase in the quantity of ACLY+ p300+ memory astrocytes in both acute and chronic EAE models, which have a stronger proinflammatory response (Lee et al., 2024). In astrocytes, proinflammatory transcription modules are expressed by GM-CSF. This mechanism is one of the causes of MS and generates pathological changes in the CNS of EAE (Wheeler et al., 2020). Additionally, astrocytes release the chemokine CXCL10, which encourages peripheral immune cell infiltration (Mills Ko et al., 2014). Astrocyte-derived IL-3 binds to IL-3Rα on microglia and peripheral myeloid cells to promote the recruitment of peripheral immune cells in MS (Kiss et al., 2023). In EAE models, astrocytic calcium channel blockade attenuates neuroinflammation and prevents demyelination (Denaroso et al., 2023).

In MS, communication between T cells and astrocytes is crucial. Astrocytes produce IL-12/IL-23 in inflammatory environments, which can amplify Th1 and Th17 reactions (Constantinescu et al., 2005). The astrocytes of mice with EAE presented increased expression levels of miR-409-3p and miR-1896, which promoted Th17 differentiation and contributed to the development of EAE (Liu et al., 2019a). However, it has also been shown that astrocyte-produced IL-27 inhibits Th17 cells (Fitzgerald et al., 2007). By triggering T-cell apoptosis via tumor necrosis factor-related apoptosis-inducing ligand-death receptor 5 (TRAIL-DR5) signaling, astrocytes in the CNS can reduce inflammation. However, under inflammatory conditions, T-cell- and microglia-produced chemicals suppress astrocyte TRAIL expression (Sanmarco et al., 2021). By producing CCL2, astrocytes stimulate glial cells and attract T cells and other immune cells, creating a positive feedback loop of inflammation. In the absence of CCL2 in astrocytes, EAE mice exhibit reduced axonal loss and demyelination, decreased diffuse activation of astrocytes and microglia in the white and gray matter, and decreased inflammation of macrophages and T cells in the white matter of the spinal cord (Kim et al., 2014).

#### Natural killer cells and neuroinflammation in multiple sclerosis

NK cells play a wide range of roles in the control of autoimmune disorders, and their modes of action are intricate and varied. NK cell proliferation in the CSF of MS patients has been demonstrated via single-cell sequencing (Straeten et al., 2022). Subsequent analysis of NK cell subgroup alterations revealed that the CSF of MS patients had more CD56bright and CD56^dim^ cells than did that of controls (Schafflick et al., 2020). The absolute number of CD56^bright^ cells and the CD56^bright^CD56^dim^ NK ratio are considerably greater in MS patients than in those with other inflammatory disorders of the CNS and noninflammatory nervous system diseases (Rodríguez-Martín et al., 2015). In MS, CD56^bright^ NK cells have been shown to play a neuroprotective role and accumulate in the periventricular area, whereas CD16^bright^ NK cells seem to be efficient cytolytic effectors (Rodríguez-Lorenzo et al., 2022).

#### B cells and neuroinflammation in multiple sclerosis

Previously, MS was believed to be an autoimmune illness mediated by T cells. The evidence indicating that B cells play a role in its pathophysiology has increased in recent years. B lymphocytes are scarce in the brains of healthy individuals, but they are greatly increased in the CSF of MS patients (Straeten et al., 2022). B lymphocytes infiltrate the perivascular area in the brain tissue of postmortem MS patients, which is associated with an earlier and more severe start (Reali et al., 2020; Moccia et al., 2022). One factor in the pathophysiology of immune-mediated disorders is the dysregulation of B-cell cytokine production. EAE develops more quickly when the autoreactive B-cell proinflammatory cytokine IL-6 is expressed at relatively high levels (Thomann et al., 2023). Proinflammatory B cells exhibit increased oxidative phosphorylation, which modulates ATP-dependent cytokine production (Li et al., 2024c). In CSF, certain chemokine patterns are linked to the presence of B-cell aggregates (Schropp et al., 2023). The B-cell chemokine CXCL13 can promote the development of ectopic lymphocytes and is increased in the serum, plasma, CSF, and active lesions of MS patients (Pan et al., 2022). Furthermore, both active and chronic inactive MS lesions have increased amounts of the CXCL12 protein, and the CXCL12 receptor CXCR4 is maintained throughout plasma cell development, which may help explain plasma cell persistence (Krumbholz et al., 2006). For certain MS patients, B-cell depletion therapy is the most effective course of treatment (Lee et al., 2021). B cells also work by releasing cytokines or interacting with T cells. Memory B cells interact with T-cell immunoglobulin and ITIM domain protein (TIGIT) on B cells and with CD155 on cTfh cells to suppress cTfh cell proliferation and IL-17 production (Asashima et al., 2022). Furthermore, the cytotoxic properties of CD8^+^ T cells can be enhanced, and granzyme B synthesis is facilitated by B-cell-expressed IL-15 (Schneider et al., 2011).

In MS, NK cells are crucial for controlling T cells. NK cells can identify and eliminate aberrant or overactivated T cells through their ability to kill them to regulate their number and condition. In the EAE animal model, NK cells eliminate similar T cells, including brain-producing, myelin antigen specific, and other types, to improve the disease (Xu et al., 2005). Granzyme K is necessary for CD56bright NK cells to target activated autologous T cells (Jiang et al., 2011). This process results in the formation of ROS, which eventually cause these autologous T cells to die. Daclizumab enhances CD56bright NK cell-mediated clearance of autoreactive T cells by binding to CD25 and upregulating granzyme K (Jiang et al., 2011). Furthermore, CD56^bright^ NK cells in the brains of MS patients exhibit increased expression of PD-L1, a hallmark of NK cell activation linked to increased NK cell cytotoxicity (Rodríguez-Lorenzo et al., 2022). Self-reactive T cells that express PD-L1 can be inhibited by PD-L1 when it binds to the coinhibitory molecule PD-1 on T cells. By inhibiting T cells, CD56^bright^ NK cells generally provide protection against MS.

#### T lymphocytes are key factors in the pathophysiology of multiple sclerosis

The immune system can correctly discriminate between “self” and “nonself” individuals to preserve immune homeostasis during normal circumstances. However, in MS patients, immune tolerance is lost, and T cells are aberrant, causing a neuroinflammatory response and targeting the CNS myelin sheath.

CD161^+^/lymphotoxin beta (LTB)^+^ T-cell populations are considered to exist in progressive MS patients with particular pathogenic T cells in the brain. The engraftment of these T cells in the CNS may begin early in the course of disease (Kaufmann et al., 2021). Eliminating CNS-homing T cells in the early stages of MS to prevent disease progression is a potential line of inquiry. One of the main genetic risk factors for MS is the human leukocyte antigen (HLA)-DR15 haplotype. By serving as an antigen-presenting structure and epitope source, HLA-DR15 enhances immunological onslaught on the CNS by encouraging the activation and growth of self-reactive T cells (Wang et al., 2020). Furthermore, the CNS of EAE mice is invaded by inflammatory group 3 innate lymphoid cells (ILC3s), which serve as APCs and encourage myelin T-cell activation (Grigg et al., 2021). The cerebral fluid of MS patients contains CNS-resident immune cells, such as CD8^+^ and CD4^+^ tissue-resident memory T cells (Ostkamp et al., 2022). These findings suggest that peripheral immune cell migration to the CNS may be the cause of MS development.

The main immune cell population in the CNS lesions of MS patients is CD8^+^ T cells, which are crucial to MS development. Numerous CD8^+^ T cells are observed in MS lesions, and it is believed that antigen presentation by the BBB encouraged the entrance of CD8^+^ T cells into the CNS (Aydin et al., 2023). Secondary progressive multiple sclerosis (SPMS) patients have increased levels of activated CD8^+^ T-cell subsets in their peripheral blood as well as increased levels of granzyme B gene expression in their CD8^+^ cells (Shi et al., 2023). In MS, peripheral immunological and metabolic alterations indicate increased proinflammatory, migratory, and activated CD8^+^ T-cell phenotypes (Kavaka et al., 2024). According to a recent study (Kurioka et al., 2016), neurological disorders may involve mucosa-associated invariant T (MAIT) cells, a subset of memory CD8^+^ T cells. When activated, MAIT cells generate IL-17 and IFN-γ, indicating that resident MAIT cells are proinflammatory and negatively impact the CNS. While there is no discernible difference in RRMS patients, the selectivity of CD8^+^ MAIT cells is decreased in the peripheral blood of primary progressive multiple sclerosis (PPMS) patients and SPMS patients (De Federicis et al., 2024). According to another study, RRMS patients have fewer MAIT cells in their blood, and this decrease is associated with the release of proinflammatory cytokines (TNF-α, IFN-γ, IL-17, and GM-CSF) (Carnero Contentti et al., 2019). However, compared with that in peripheral blood, the fraction of MAIT cells in CSF was much greater, suggesting that MAIT cells may be able to migrate and breach the BBB (Carnero Contentti et al., 2019). The ability of MAIT cells to cross the BBB and release proinflammatory cytokines in the brain was also demonstrated by immunofluorescence staining of postmortem brain tissue from SP-MS patients (Gargano et al., 2022).

In older patients with MS, there is an aberrant increase in CD4^+^ T cells, activation, and cytotoxic effect characteristics (Zuroff et al., 2022). Peripheral CD4^+^ T-cell IL-7Rα is crucial for the immunological response, pathogenesis, and CD4^+^ T-cell maintenance in an EAE mouse model (Azizi et al., 2024). When CD4^+^ T cells lose the *IL-7Rα* gene, their numbers gradually and significantly decrease. The number of Tregs also decreases, although not as much as those of other CD4^+^ T cells (Azizi et al., 2024). Compared with CD4^+^c-Met^−^ T cells, CD4^+^ T cells from EAE model mice present greater expression of the cellular mesoderm epithelial transition factor (c-Met) and higher levels of Integrin α4 (Itgα4) (Benkhoucha et al., 2022). The primary function of Itgα4 is to attract inflammatory cells to the CNS (Engelhardt et al., 1998). A key factor in MS pathophysiology is thought to be the increased proinflammatory phenotype of CD4^+^c-Met^+^ T cells, which are associated with Th1 and Th17 polarization (Benkhoucha et al., 2022). Itgα4 is substantially expressed in CD4^+^c-Met^+^ T cells, and an increase in CD4^+^c-Met^+^ T cells is also observed in the peripheral blood or CNS of MS patients (Benkhoucha et al., 2022). In mouse models of EAE, Rel-A, a member of the NF-κB family, facilitates the transformation of CD4^+^ conventional T cells into Th17 cells (Lalle et al., 2024). The transcription factor early growth response protein 2 (EGR2) stimulates CNS recruitment and Th17 cell differentiation in a manner that is dependent on RORγt (Gao et al., 2023).

The function of CD8^+^ Tregs is linked to disease severity in MS patients (Benallegue et al., 2022). One type of regulatory cell that either directly or indirectly controls T cells is CD28^−^CD8^+^ T cells. This cell type has been shown to reduce the severity of MS in animal models (Chen et al., 2018). The activity of histone deacetylase 7 (HDAC7) contributes to T-cell activation, differentiation, and death. HDAC7 is crucial for preserving peripheral Treg function since its absence or aberrant function in Tregs may limit their ability to suppress other cells. Neuroinflammation is more severe in HDAC7 knockout mice (Axisa et al., 2022).

In summary, T lymphocytes play a complicated and significant role in the neuroinflammatory response associated with MS, and their control may offer novel approaches for MS treatment.

**Figure 4:**
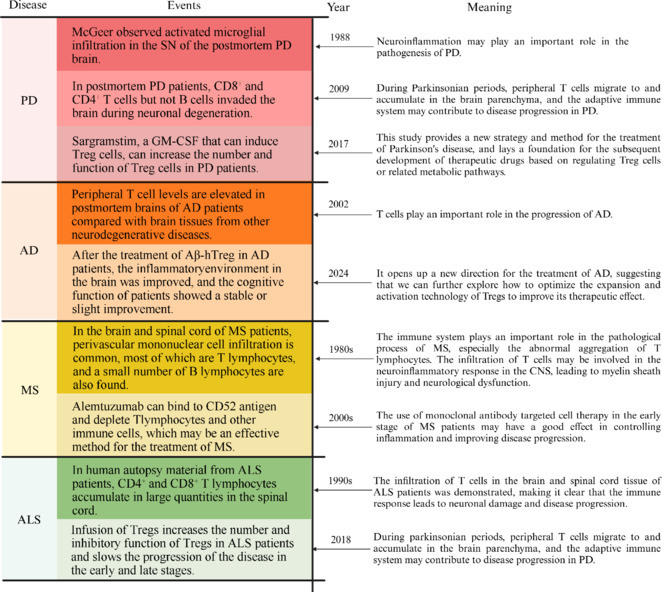
Mapping of milestone events in T-cell-induced neuroinflammation in NDD patients. Aβ-hTreg: Amyloid beta-specific human regulatory T cells; AD: Alzheimer’s disease; ALS: amyotrophic lateral sclerosis; CNS: central nervous system; GM-CSF: granulocyte‒macrophage colony-stimulating factor; MS: multiple sclerosis; NDD: neurodegenerative disease; PD: Parkinson’s disease; SN: substantia nigra.

Finally, we summarize the key milestone events in the study of T cells in NDD (**Figure 4**). By combining the important nodes in the timeline, we can clearly see the progress from the discovery of T cells and disease associations to the exploration of targeted T-cell therapy for NDD. These events have provided clues for researchers to understand NDD pathology, drive therapeutic innovations based on targeted T cells, and have important implications for improving disease progression.

## Clinical Transformation

As scientists continue to investigate the function of the immune system in the pathogenesis of NDD, T cells have emerged as important factors. As a result, treatment approaches that target T cells have steadily gained popularity in medical research. Through the use of T cells, researchers expect to improve neuroinflammation in NDD, as well as the course and prognosis of patients’ illnesses.

### Enhancing the function of Tregs

Numerous techniques have been devised to increase Tregs *in vivo* to enhance the immunological environment of NDD because it is believed that Tregs inhibit neuroinflammation and protect neurons. Treg expansion drugs include low-dose IL-2, Sargramostim, rapamycin, and anti-CD28 monoclonal antibodies.

Sargramostim is an immunological regulator that can restore the body’s immune status to its baseline level. Sargramostim was found to be safe and well tolerated in a double-blind, placebo-controlled phase Ia clinical trial, which also revealed a rise in Treg levels compared with those in placebo controls. Despite the safety and tolerability of this treatment, minor adverse effects, such as injection site responses, bone and limb pain, and increased white blood cell counts, have been recorded (Gendelman et al., 2017). Thus, low dosages of well-tolerated Sargramostim were used to treat PD patients in a phase Ib clinical trial. Tregs are greatly expanded in both number and function, which is beneficial and crucial for preserving the body’s immunological balance, reducing the overabundance of immune responses, and assisting in the restoration of immune homeostasis throughout the body (Olson et al., 2021a). Tregs have thus demonstrated great promise in the management of PD.

Aldesleukin is a recombinant human IL-2 drug, and currently, there is a single ongoing phase II/III clinical trial for ALS (Camu et al., 2020). Compared with a placebo, aldesleukin dramatically increased the number of Tregs in a phase II clinical trial including 36 ALS patients (Camu et al., 2020). Treg infusion was safe and well tolerated in all patients in the phase I clinical study of autologous transplantation of Tregs in ALS patients. Additionally, the number and inhibitory function of Tregs increase, slowing the course of the early and late phases of the disease (Thonhoff et al., 2018). Dimethyl fumarate activates nuclear factor (erythrocyte-derived 2), resulting in anti-inflammatory and cell protective effects (Scannevin et al., 2012). Dimethyl fumarate suppressed proinflammatory T cells and increased Treg counts. However, dimethyl fumarate treatment for ALS does not increase patient survival, according to data from phase II clinical trials (Vucic et al., 2021).

According to the results of IIa clinical trials, the Treg cell counts of MS patients can be safely and efficiently increased with low-dose IL-2 (Barde et al., 2024). Furthermore, IFN-β1a, rapamycin, and nanocurcumin help MS patients regain their Treg function and frequency (Bagherpour et al., 2018; Ebrahimimonfared et al., 2018; Dolati et al., 2019).

### Monoclonal antibodies

A common treatment for RRMS is alemtuzumab, a humanized monoclonal antibody that targets CD52. Alemtuzumab treatment caused the immune system of RRMS patients to shift from a proinflammatory condition to an anti-inflammatory condition. In particular, the percentage of Treg cells in the peripheral blood increased, while the levels of the anti-inflammatory cytokines IL-10 and TGF-β1 increased, and the levels of the proinflammatory cytokines IL-17 and IFN-γ sharply decreased (De Mercanti et al., 2016). Alemtuzumab’s treatment strategy against RRMS is linked to lymphocyte reproliferation, which favors the regulatory subsets found in T cell, B cell, and NK cell compartments, according to a clinical trial (Gilmore et al., 2020). Although alemtuzumab has demonstrated outstanding results in a number of clinical trials, it also has certain negative effects, including an increased risk of infection and the potential to cause the development of autoimmune disorders (Ruck et al., 2018).

At present, rituximab, ocrelizumab, ofatumumab, and ublituximab are the four types of anti-CD20 monoclonal antibodies used to treat MS. Rituximab depletes B cells by binding to the CD20 antigen on their surface. Although rituximab has not yet received official approval to treat MS, it has demonstrated some application value and is frequently used to treat RRMS in clinical settings. Rituximab has the ability to decrease the number of CD20^+^ T cells in addition to B cells (Palanichamy et al., 2014). In the pathophysiology of MS, CD20^+^ T lymphocytes have significant proinflammatory functions (Arneth, 2024). Research has shown that, following rituximab treatment, the majority of MS patients have fewer B and T lymphocytes in their CSF (Cross et al., 2006). Furthermore, peripheral blood CD4^+^ and CD8^+^ T cells dramatically decrease the proinflammatory response, particularly the two proinflammatory response types Th1 and Th17 (Bar-Or et al., 2010). These changes are often regarded as secondary consequences of B-cell depletion on pathogenic T cells. In March 2017, the US Food and Drug Administration (FDA) authorized ocrelizumab, a medication that specifically targets B cells, for the treatment of MS. The likelihood of disability progression is generally reduced for MS patients with increased serum levels of ocrelizumab (Hauser et al., 2023). During treatment, CD20^+^ T cells, rather than just B cells, are included in the object of action (Gingele et al., 2018). Furthermore, following ocrelizumab treatment, the initial/effector subset ratios of CD4^+^ and CD8^+^ T cells decreased, favoring naive cells over effector cells. Moreover, the quantity of IFN-γ-producing CD4^+^ and CD8^+^ T cells decreases (Fernández-Velasco et al., 2021). The main purpose of ubituximab, a glycosy-engineered anti-CD20 monoclonal antibody, is to treat MS patients. According to one study, ublituximab reduces the number of CD20^+^ T cells in MS patients. The overall T-cell lineage changes toward an anti-inflammatory phenotype, and the percentage of Tregs increases (Lovett-Racke et al., 2021). These modifications suggest that ublituximab may be able to control potentially harmful CD4^+^ T cells in MS.

### Small-molecule T-cell inhibitors

NDD can be treated with small-molecule T-cell inhibitors, which can control the activity of T-cell subsets and prevent neuroinflammation. In animal models of NDD, cyclosporine A and tacrolimus (FK506), two traditional T-cell inhibitors, have been demonstrated to have potential therapeutic effects. They both work similarly to prevent T-cell activation by blocking calcineurin (Abecassis et al., 1988; Umland et al., 1999). Cyclosporine A and FK506 are believed to strongly depress cellular immunity by blocking immune cell activation and T-cell IL-2 production (O’Keefe et al., 1992). Cyclosporine A effectively alleviated motor impairments and coordinated the loss of SN dopaminergic neurons in a rotenone-induced PD model in rats (Singh et al., 2022). In MPTP-injured animals, Tamburrino et al. demonstrated that cyclosporine A can decrease the amount of nuclear factor of activated T cells and cytoplasmic 3 (NFATc3) in activated T cells and reduce mitochondrial stress in the midbrain, which has an anti-inflammatory effect and effectively enhances the ability of the animals to exercise (Tamburrino et al., 2015). FK506 may prevent dopaminergic neuron degeneration in PD animal models via α-syn by controlling immunological responses and lowering inflammatory cell infiltration (Van der Perren et al., 2015). The prevalence of dementia, including AD, can be considerably decreased by both tacrolimus and cyclosporine A, with tacrolimus having a stronger effect (Silva et al., 2023). Furthermore, although cyclosporine A has demonstrated some effectiveness in treating MS, its general use is restricted due to its adverse effects (Rudge, 1988). Through the inactivation of inflammatory cells, oral tacrolimus can prevent autoimmunity in the pathophysiology of MS (Kim et al., 2017).

Sphingosine 1-phosphate receptor (S1PR) modulators are a type of oral medication with a distinct mechanism of action. By preventing lymphocytes from leaving the lymph nodes and thymus, S1P receptor modulators decrease the number of lymphocytes in the circulation and decrease the infiltration of inflammatory cells into the CNS. Several S1P receptor modulators, including fingolimod, siponimod, ozanimod, and ponesimod, have been used extensively to treat MS (Fronza et al., 2021). Fingolimod was the first oral medication created and authorized for the treatment of RRMS in 2010 (Chun and Brinkmann, 2011). Siponimod was authorized by the FDA in March 2019 to treat active MS (Al-Salama, 2019). Ozanimod capsules were authorized by the FDA in March 2020 to treat individuals with relapsing forms of MS (Lamb, 2020). In 2021, the FDA authorized ponesimod for the treatment of adult MS (Alnaif et al., 2023).

The mechanism of action of these small-molecule T-cell inhibitors for the treatment of NDD is quite clear, and clinical trials have gone well. However, long-term use of this class of inhibitors may have negative effects on the immune system, and systemic immunosuppression of T cells may disrupt normal immunological function. Thus, more research is needed to confirm the long-term safety and tolerance of this inhibitor class.

### Chimeric antigen receptor T-cell immunotherapy

Chimeric antigen receptor T-cell (CAR-T) immunotherapy refers to the genetic modification of T cells to target specific antigens for immunotherapy. CAR-T-cell treatment has shown exceptional effectiveness in treating hematological malignancies, but its use in NDDs has been limited due to the cytotoxic potential of CD8^+^ T cells (Boskovic et al., 2024). De Paula Pohl et al. (2020) used a mouse model of EAE to genetically modify Tregs to express cars that target myelin proteins. The severity of the disease, number of demyelinating lesions, and degree of inflammatory cell infiltration in the brain and spinal cord were considerably decreased by this CAR-Treg treatment. Consequently, CAR-T-cell therapy has demonstrated a wide range of potential uses in the management of MS. It is anticipated that CAR-T-cell therapy will play a significant role in treating NDD as a result of ongoing technological advancements and expanding clinical trials.

Targeting T cells in various ways to treat NDD offers encouraging but challenging prospects (**Table 1**). A deeper comprehension of T-cell processes in NDD holds promise for overcoming current obstacles and developing more potent treatments for NDD patients in the future.

**Table 1:** Comparison of different approaches to target T cells for the treatment of NDD

Method	Mechanism	Application	Advantage	Challenge	Reference
Treg therapy	Autologous or allogeneic transplantation of Tregs or stimulation of Treg expansion with the use of signals such as IL-2.	PD, ALS, MS.	(1) It has dual effects of neuroprotection and immunomodulation. (2) It can be achieved through a variety of pathways, such as cell therapy or IL-2 therapy.	(1) Polyclonal Tregs may cause systemic immunosuppression and increase the risk of infection. (2) The development and application of disease-specific Tregs is still in the research stage.	Gendelman et al., 2017; Bagherpour et al., 2018; Ebrahimimonfared et al., 2018; Thonhoff et al., 2018; Dolati et al., 2019; Camu et al., 2020; Olson et al., 2021a; Barde et al., 2024
Antibody-mediated T-cell depletion	Monoclonal antibodies are used to specifically bind key molecules on the surface of T cells to block the activation signal of T cells or regulate their function.	MS	(1) High specificity; it can accurately act on T-cell-related targets (2) The mechanism of action is clear, and targeted antibodies can be designed according to the target.	(1) It may cause an immunogenic reaction, leading to the body's immune response to the monoclonal antibody. (2) Long-term use may affect normal immune function, and there are potential off-target effects	Cross et al., 2006; Palanichamy et al., 2014; De Mercanti et al., 2016; Gingele et al., 2018; Ruck et al., 2018; Gilmore et al., 2020; Fernández-Velasco et al., 2021; Lovett-Racke et al., 2021; Hauser et al., 2023
Small molecule T-cell inhibitors	Inhibition of T-cell activation and migration.	AD, PD, MS	There are clinical drugs that can be rapidly transformed.	Systemic immunosuppression of T cells may interfere with normal immune function.	Abecassis et al., 1988; Rudge, 1988; O'Keefe et al., 1992; Umland et al., 1999; Chun and Brinkmann, 2011; Tamburrino et al., 2015; Van der Perren et al., 2015; Kim et al., 2017; Al-Salama, 2019; Lamb, 2020; Fronza et al., 2021; Singh et al., 2022; Alnaif et al., 2023; Silva et al., 2023
CAR-T	Engineered T cells target pathological proteins.	MS	(1) Highly targeted, reducing damage to normal tissues. (2) Specific receptors can be designed according to the characteristics of the disease.	(1) CAR-T cells are difficult to penetrate the BBB and enter the CNS. (2) Overactivation of CAR-T cells may lead to excessive immune response.	De Paula Pohl et al., 2020; Boskovic et al., 2024

AD: Alzheimer's disease; BBB: blood–brain barrier; CAR-T: chimeric antigen receptor T-cell immunotherapy; CNS: central nervous system; IL-2: interleukin-2; MS: multiple sclerosis; NDD: neurodegenerative disease; PD: Parkinson's disease; ALS: amyotrophic lateral sclerosis; Treg: regulatory T.

## Limitations

The function of T-cell subpopulations, the variety of interactions with various subpopulations, the complexity of the disease, the constraints of clinical samples and animal models, and other factors make studying T cells in NDD challenging.

The pathophysiology of NDD is extremely complicated and includes environmental and genetic variables and the immune condition of the body. T cells are merely one component of NDD, and it is challenging to determine exactly how T cells contribute to the pathophysiology of NDD. Furthermore, NDD typically has a protracted duration. The immune cells and molecular pathways involved vary depending on the stage of the disease and the pathophysiological alterations of the patients. This presents a significant obstacle to NDD research. Directly observing and interacting with T cells in the CNS is challenging because of the BBB. Additionally, the CNS has a wide variety of cell types and cell-to-cell interactions. Neurons, glial cells, and immune cells all have intricate regulatory and communication systems. In the future, to explore NDD in depth, researchers need to comprehensively consider how genetic, environmental, and immune factors drive the occurrence and development of NDD. In addition, in future investigations, emerging technologies such as single-cell sequencing and spatial transcriptomics should also be adopted to obtain the gene expression profiles of various T-cell subsets for a comprehensive molecular description of NDD. Pathogenic genes, the current environment and immune conditions were combined to observe changes in disease progression and the T-cell-mediated neuroinflammatory response. Gene editing subsequently treats NDD by targeting genes that are overactive or silenced during illness initiation and progression. Gene editing methods include early RNA interference, zinc finger nucleases (ZFNs), transcription activator-like effector nuclease (TALEN) and, more recently, clustered regularly interspaced short palindromic repeats-associated protein 9 (CRISPR-Cas9). Owing to its ability to modify particular gene sequences, CRISPR-Cas9 shows promise in the treatment of NDD (Luo et al., 2019). To decrease neuroinflammation and alter the equilibrium of the immune milieu, CRISPR-Cas9 can be utilized to instruct T cells or control T-cell-mediated neuroinflammation pathways both *in vivo* and *in vitro*. These findings provide a rationale for the development of integrated prevention and treatment strategies.

Research on T cells in NDD patients and animal models has certain drawbacks. First, it can be difficult to obtain suitable T-cell samples from patients. The majority of research on T cells in patients has focused only on peripheral blood. T-cell alterations in peripheral blood may not accurately represent the condition of T cells in the brain of NDD patients because of the BBB. T-cell collection in the brain is challenging, the sample size is small, and dynamic observation throughout illness progression is challenging. However, individual characteristics, such as age, sex, underlying conditions, and medication history, make it challenging to assess the sample’s representativeness and the reliability of the findings. Animal models of NDD serve as the basis for a large portion of current research, yet it is well known that human disease and animal models are never exactly the same. The translation of research findings from animal models to clinical applications is limited because the response of T cells in animal models might not accurately reflect the actual circumstances of NDD patients.

There are several subgroups of T cells, including Th, CTL, and Treg cells. These subgroups have multiple functions in the NDD and can be further split. The majority of T-cell subsets play two roles: they contribute to tissue healing to some degree in addition to secreting inflammatory factors that enhance neuroinflammation. Furthermore, these subgroups interact in intricate ways. Furthermore, T cells have reciprocal regulatory effects on other immune cells, including B cells, astrocytes, and microglia. This makes accurate understanding of the overall function of T cells more difficult and presents challenges for targeted T-cell therapy.

Future research is needed to examine the features of T-cell‒immune cell interactions in NDD patients or animal models at various phases of the disease via longitudinal monitoring. Comprehending the dynamic alterations in T cells during the course of illness development will facilitate the implementation of suitable immune intervention strategies at various disease stages and enable precision treatment. In addition, studies must compare various subtypes of NDD thoroughly, examine the functional traits of immune cells within each subtype, and identify potential therapeutic targets and biomarkers to aid in the development of novel medications.

In conclusion, there have been some advancements in the current study of T cells in NDD, but there are still numerous obstacles to overcome. To better understand the function and mechanism of T cells in NDD and offer a more potent foundation for NDD treatment, future research must constantly advance its methodologies and technological capabilities.

## Conclusion

The pathophysiology of NDD is influenced by neuroinflammation, which causes neurons to degenerate and the disease to advance. The balance between immune cell subpopulations is disrupted, and damage to host tissues leads to pathological diseases. The primary factors that accelerate NDD are the activation of microglia and astrocytes, as well as the disruption of B cells and T cells induced by NDD neuroinflammation. The key elements of the adaptive immune system are B cells and T cells. Importantly, T-cell population homeostasis plays a significant role in the maintenance and management of neuroinflammation.

Various studies have reported different T-cell populations in the human CNS and blood, and changes in these cell populations play important roles in NDD neuroinflammation (**Table 2**). CD8^+^ T cells play a significant role in NDD. Through the breached BBB, they can enter the brain, activate glial cells, produce cytotoxic substances, and cause other inflammatory reactions that might speed up neuronal death and worsen the disease’s pathological changes and symptoms. The involvement of CD4^+^ T lymphocytes in NDD is intricate and vital. On one hand, CD4^+^ T cells work to control the immune response and preserve the equilibrium of the immunological milieu in the CNS, both of which contribute to neuroprotection. On the other hand, CD4^+^ T cells can be overactivated and abnormally differentiated, which can lead to the production of numerous inflammatory factors, worsen neuroinflammation, attract more peripheral immune cells to the CNS, and either directly or cooperatively damage neurons to accelerate the progression of NDD. Th17 cells are well-known proinflammatory CD4^+^ T cells, and breakdown of the BBB in NDD patients creates conditions for Th17 cells to enter the brain. However, Tregs have a protective effect on NDD patients. Therefore, Tregs have demonstrated considerable promise for use in the treatment of NDD.

**Table 2:** Mechanisms by which T-cell subsets regulate NDD neuroinflammation

Disease	T-cell subtype	Influence	Potential mechanism	Reference
PD	CD8^+^ T cells	Accelerate α-syn aggregation and neuronal death, positively correlate with autonomic dysfunction and psychiatric comorbidities	Secrete high levels of TNF-α and IFN-γ cytokines	Galiano-Landeira et al., 2020; Bhatia et al., 2021; He et al., 2021; Wang et al., 2022; Capelle et al., 2023
	Th1	Promote inflammation	Secrete pro-inflammatory cytokines	Kustrimovic et al., 2018
	Th2	Preventdopaminergic neuron death	Prevent Th1 cells from proliferation and secrete anti-inflammatory cytokines	Arimoto et al., 2007; Yan et al., 2021
	Th17	Increase BBB permeability, promoteinflammation and damageneurons	Increase cytokine release, enhance the release of TNF-α on microglia and express granzyme B; LFA-1 expressed on Th17 cells binds to ICAM-1 in dopamine-related neurons	Kebir et al., 2007; Reynolds et al., 2010; Liu et al., 2016; Dutta et al., 2019; Liu et al., 2019b; Sommer et al., 2019; Gate et al., 2021; Li et al., 2023b
	Tregs	Slow the degeneration of DA neurons and prevent the progressive development of neuroinflammation in animal models of PD.	Decrease CD4^+^ and CD8^+^ T-cell counts, Th17 immune response, microglia activation	Reynolds et al., 2007, 2010; Huang et al., 2019; Olson et al., 2020, 2021b; Badr et al., 2022; Park et al., 2023
AD	CD8^+^ T cells	Promote neuroinflammation	Activate microglia	Jorfi et al., 2023; Kimura et al., 2024; Yamakawa and Rexach, 2024
	Th1	Promote inflammation	Release IFN-γ, which causes inflammation, and direct T cells to the site of inflammation	Fatemi Langroudi et al., 2023; Vasantharekha et al., 2024
	Th17	Promote inflammation	Promote the release of inflammatory factors and induce the activation of microglia	Brigas et al., 2021; Machhi et al., 2021; Hao et al., 2024
	Tregs	Delay disease progression of AD-like pathology	Downregulated proinflammatory factor, complement cascade, activation of microglia, and inhibition of astrocyte reactivity	Faridar et al., 2022; Stym-Popper et al., 2023; Zuo et al., 2024
ALS	CD8^+^ T cells	Lead to neurological dysfunction and accelerate disease progression	Secrete more granzyme B and IFN-γ	Campisi et al., 2022; Kaur et al., 2022; Yildiz et al., 2023
	CD4^+^ T cells	Protective effect, reduce the severity of clinical conditions, and related to cognitive impairment	Maintain the balance between cytotoxicity and nutrition of glial cells and play a neuroprotective role	Beers et al., 2017; Yang et al., 2021b; Yildiz et al., 2023
		The late stage shows a proinflammatory phenotype	Induce DNA damage	Campisi et al., 2022; Lu et al., 2024; Zaccai et al., 2024
	Th1	Promote inflammation	Secrete pro-inflammatory cytokines	Italiani et al., 2014
	Th2	Be correlated with the risk of death	Secrete anti-inflammatory cytokines	Jin et al., 2020
	Th17	Be related to the severity of the disease	Secrete pro-inflammatory cytokines	Jin et al., 2020; Limone et al., 2024
	Tregs	Reduce neuroinflammation, prolong the lifespan of ALS mouse models, and be correlated with the rate of disease progression in patients	Increase the expression of neurotrophic factor gene in the spinal cord and peripheral nerves of ALS animals, and decrease the immune reactivity of microglia and astrocytes in the spinal cord of ALS animals	Sheean et al., 2018; Rajabinejad et al., 2020; Yazdani et al., 2022
MS	CD8^+^ T cells	Promote neuroinflammation, lead to myelin loss and nerve damage	High expression of granzyme B and secretion of pro-inflammatory cytokines (such as IL-17 and IFN-γ)	Carnero Contentti et al., 2019; Shi et al., 2023; De Federicis et al., 2024; Kavaka et al., 2024
	CD4^+^ T cells	Promote neuroinflammation.	Tend to be Th1 and Th17 polarized	Benkhoucha et al., 2022
	Tregs	Reduce the severity of MS	Direct or indirect control of T-cell activation	Benallegue et al., 2022

AD: Alzheimer's disease; ALS: amyotrophic lateral sclerosis; BBB: blood–brain barrier; DA: dopamine; ICAM-1: intercellular adhesion molecule-1; IFN-γ: interferon-gamma; IL-17: interleukin-17; LFA-1: lymphocyte function-associated antigen-1; MS: multiple sclerosis; PD: Parkinson's disease; Th1: T helper cell 1; Th2: T helper cell 2; Th17: T helper cell 17; TNF-α: tumor necrosis factor-alpha; Treg: regulatory T; α-syn: alpha-synuclein.

T cells mediate neuronal death and promote NDD through a variety of different pathways. However, the current understanding of how each subpopulation of T cells in NDD patients responds to neuroinflammation is unclear. Thus, elucidating the relationships of various cells and cytokines with T cells is crucial. Identifying better strategies to control the etiology of NDD may be possible by elucidating the involvement of T cells in NDD, which may lead to new therapeutic strategies. Furthermore, additional research on T-cell regulation in NDD is warranted in the future.

## Data Availability

*Not applicable*.
